# Regulatory B cells improve ventricular remodeling after myocardial infarction by modulating monocyte migration

**DOI:** 10.1007/s00395-021-00886-4

**Published:** 2021-07-24

**Authors:** Jiao Jiao, Shujie He, Yiqiu Wang, Yuzhi Lu, Muyang Gu, Dan Li, Tingting Tang, Shaofang Nie, Min Zhang, Bingjie Lv, Jingyong Li, Ni Xia, Xiang Cheng

**Affiliations:** 1 Department of Cardiology, Union Hospital, Tongji Medical College, Huazhong University of Science and Technology, Wuhan, 430022 China; 2 Key Laboratory for Biological Targeted Therapy of Education Ministry and Hubei Province, Union Hospital, Tongji Medical College, Huazhong University of Science and Technology, Wuhan, 430022 China; 3 Department of Pediatrics, Union Hospital, Tongji Medical College, Huazhong University of Science and Technology, Wuhan, 430022 China

**Keywords:** Myocardial infarction, Ventricular remodeling, Regulatory B cells, Interleukin 10, Monocytes

## Abstract

Overactivated inflammatory responses contribute to adverse ventricular remodeling after myocardial infarction (MI). Regulatory B cells (Bregs) are a newly discovered subset of B cells with immunomodulatory roles in many immune and inflammation-related diseases. Our study aims to determine whether the expansion of Bregs exerts a beneficial effect on ventricular remodeling and explore the mechanisms involved. Here, we showed that adoptive transfer of Bregs ameliorated ventricular remodeling in a murine MI model, as demonstrated by improved cardiac function, decreased scar size and attenuated interstitial fibrosis without changing the survival rate. Reduced Ly6C^hi^ monocyte infiltration was found in the hearts of the Breg-transferred mice, while the infiltration of Ly6C^lo^ monocytes was not affected. In addition, the replenishment of Bregs had no effect on the myocardial accumulation of T cells or neutrophils. Mechanistically, Bregs reduced the expression of C–C motif chemokine receptor 2 (CCR2) in monocytes, which inhibited proinflammatory monocyte recruitment to the heart from the peripheral blood and mobilization from the bone marrow. Breg-mediated protection against MI was abrogated by treatment with an interleukin 10 (IL-10) antibody. Finally, IL-10 neutralization reversed the effect of Bregs on monocyte migration and CCR2 expression. The present study suggests a therapeutic value of Bregs in limiting ventricular remodeling after MI through decreasing CCR2-mediated monocyte recruitment and mobilization.

**Supplementary Information:**

The online version contains supplementary material available at 10.1007/s00395-021-00886-4.

## Introduction

Advances in the therapy of myocardial infarction (MI) by successful restoration of the occluded coronary artery profoundly decrease the mortality rate. However, ischemic heart disease, which is the main cause of heart failure, is becoming a major public health concern all over the world [41]. Thus, intensive efforts have been targeted at left ventricular pathological remodeling. Ventricular remodelling relates to the changes of cardiac function and structure in response to multifactorial stimuli, involving myocytes, interstitial cells and interstitial matrix [17]. Both clinical and experimental studies suggest that inflammatory responses participate in ventricular remodeling post-MI, attempting to clear up matrix debris and dead cells. However, overactive inflammatory signaling exacerbates myocardial injury and causes adverse cardiac remodeling. Thus, timely restraint of the excessive and pathological inflammatory responses is essential in promoting tissue repair and preserving cardiac function [11, 18].

Recently, a distinct B cell subset reported to suppress inflammation and modulate immune responses has been named as regulatory B cells (Bregs). Bregs lack specific surface markers or transcription factors equivalent to forkhead-box-P3 (Foxp3) in regulatory T cells (Tregs), with different phenotypes occurring in a disease-dependent manner. B cells which are able to produce interleukin 10 (IL-10) upon stimulation, are the best-characterized Bregs, with IL-10 as both a defining marker and functional cytokine [5, 23, 40]. Multiple studies support the protective roles of Bregs in diseases through secreting immunomodulatory cytokines [5, 23, 40, 48]. Breg-derived IL-10, the most frequently investigated, inhibits T cell differentiation into proinflammatory cell subtypes, such as Th1 and Th17 cells, while it promotes T cell differentiation into anti-inflammatory cell subtypes, such as Tregs [32, 36, 45, 52]. In addition, Bregs also suppress the secretion of proinflammatory cytokines by monocytes and dendritic cells via the IL-10-dependent mechanism [40]. In addition to IL-10, Breg-derived transforming growth factor β1 (TGF-β1) and interleukin 35 (IL-35) also have been demonstrated to mediate the protective roles of Bregs against dextran sodium sulfate-colitis, experimental autoimmune encephalomyelitis, and experimental uveitis [39, 42, 47].

Previous studies reported that Bregs played important roles in the autoimmune diseases, infections, cancer, and organ transplantation [5, 23, 40, 48]. A recent research showed that IL-10-producing B cells, abundant in the pericardial adipose tissue, infiltrated into the infarcted heart, facilitated the alleviation of inflammation, and reduced myocardial injury after MI. This study identified the role of endogenous Bregs in MI [49]. However, this raises an important question regarding whether exogenous Bregs have therapeutic effects on MI, which would be helpful in developing Breg therapy for repairing the infarcted heart.

The present study provided clear evidence that transferred Bregs improved cardiac function and ameliorated ventricular remodeling post-MI by down-regulating the expression of C–C motif chemokine receptor 2 (CCR2) in monocytes, thus leading to attenuated infiltration of proinflammatory monocytes into the infarcted myocardium. In addition, we demonstrated that the characteristic cytokine IL-10 was critical for Breg-induced cardiac protection in MI. Of clinical relevance, these findings revealed a therapeutic role of Bregs in improving clinical outcomes post-MI.

## Methods

Detailed experimental methods are presented in the Supplementary material.

### Mice

8–12-week-old male C57BL/6J mice and CD45.1 mice with a C57BL/6J background were purchased from Beijing Vital River Laboratory Animal Technology (Beijing, China). IL-10-GFP (green fluorescent protein) knock-in mice (Stock No. 014530) which had a C57BL/6J background were obtained from the Jackson Laboratory (Bar Harbor, USA). IL-10 knock-out (KO) mice on a C57BL/6J background were purchased from Bioray Laboratories (Shanghai, China). The mice were kept in the Tongji Medical School Animal Care Facility. They were maintained on a chow diet at 25 °C in a 12-h light/12-h dark environment. All of our animal studies were carried out conformed to the National Institutes of Health guidelines and were authorized by the Animal Care and Utilization Committee of Huazhong University of Science and Technology, China.

### In vivo MI protocol

MI model was induced by permanent ligation of the left anterior descending coronary artery (LAD) as described previously with minor modifications [44]. In brief, the mice were anesthetized by injection of ketamine (50 mg/kg) and pentobarbital sodium (50 mg/kg) intraperitoneally, orally intubated and connected to a rodent respirator. A left thoracotomy was performed, and the LAD was ligated using a 6–0 suture. In the sham group, mice underwent the same surgical procedures except that the suture passed under the LAD was not tied.

### Treatment and groups

In the adoptive transfer experiments, MI mice were randomized to receive 2 × 10^6^ purified IL-10^+^ B cells (Breg group), 2 × 10^6^ IL-10^−^ B cells (control B cell group), or an equal volume of phosphate buffered saline (PBS group) by intravenous injection immediately after the operation. C57BL/6J mice in the sham group were the controls. Cardiac function and structural remodeling were assessed on day 28 post-MI. Neutrophils, T cells and macrophages that infiltrated into the heart were detected by flow cytometry 3 or 7 days after MI. Monocytes in the spleen, bone marrow, blood and heart and their CCR2 expression in the above tissues, except for the heart, were also measured 1 and 3 days after MI.

To determine the functional molecule of Bregs in MI, MI mice were randomly assigned for treatment with the anti-IL-10, anti-TGF-β1, anti-IL-35 or isotype control antibodies. For the IL-10 and TGF-β1 blockade, 200 µg anti-IL-10 (clone JES5-16E3, eBioscience, USA), anti-TGF-β1 (clone 1D11.16.8, BioXcell, USA) or isotype-matched (Rat IgG2b, clone RTK4530, Biolegend, USA; Mouse IgG1, clone MOPC-21, BioXcell, USA) control antibodies were intraperitoneally administered to the mice on day 0 and day 7 after MI [10, 12, 24, 26, 28, 50]. In the IL-35 blocking experiments, mice were administrated with the anti-EBI3 (Epstein–Barr virus-induced gene 3, a subunit of IL-35, clone V1.4C4.22, Merck Millipore, USA) or isotype-matched (Mouse IgG2b, clone MPC-11, BioXcell, USA) control antibody at an initial dose of 100 µg/mouse on day 0 and an additional dose of 50 µg on day 7 [20, 46]. Cardiac function and structural remodeling were assessed on day 28 post-MI. In addition, the MI mice were administered with Bregs in the presence of the anti-IL-10 or isotype control antibody, and the numbers of monocytes and their subsets that infiltrated into the heart at day 1 and day 3 after MI were detected by flow cytometry. CCR2 expression in monocytes from the bone marrow, spleen and blood was also measured 1 and 3 days after MI.

### Statistics

Data are presented as means ± SEM. To assess the statistical significance, Shapiro–Wilk test was first used to determine the data normality, and equal variance was analyzed between two groups by the F test and by Bartlett's test for multiple comparisons. If the evaluation passed, differences were evaluated using two-tailed unpaired Student’s *t* test between two groups and one-way ANOVA for multiple comparisons followed by Tukey’s post hoc test. Otherwise, Mann–Whitney *U* test or Kruskal–Wallis test with Dunn’s multiple comparisons test was performed. Survival distributions were estimated by the Kaplan–Meier method and compared by log-rank test. All analyses were performed using GraphPad Prism 8.3.0 (Graph Pad Prism Software, USA), and *P* < 0.05 was considered to be statistically significant.

## Results

### Breg transfer attenuates post-MI cardiac dysfunction and adverse ventricular remodeling

**Fig. 1:**
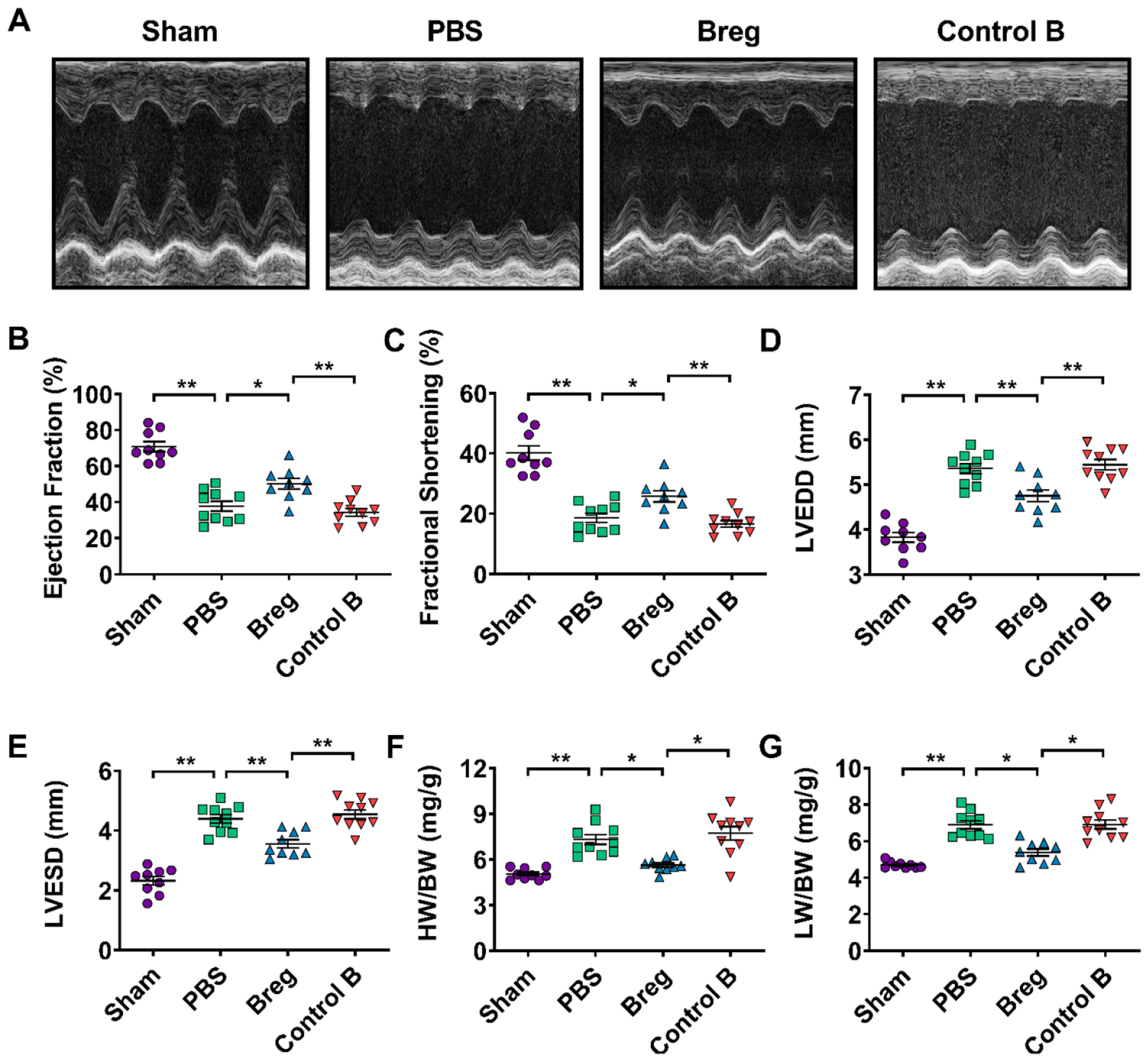
Adoptive transfer of Bregs improves cardiac function after MI. **a** Representative M‐mode echocardiographic images of the left ventricle 28 days after MI. **b**–**e** Analysis of ejection fraction (**b**), fractional shortening (**c**), LVEDD (**d**) and LVESD (**e**) by echocardiography at day 28 after MI. *n* = 9–10 per group. **f**, **g** HW/BW and LW/HW were measured at day 28 after MI. *n* = 9–10 per group. Data are expressed as means ± SEM. *P < 0.05, **P < 0.01. Data in **b**–**e** were analyzed by one-way ANOVA, followed by Tukey’s post hoc test. Data in **f** and **g** were analyzed by Kruskal–Wallis test with Dunn’s multiple comparisons test. *Sham* sham-operated group, *PBS* MI mice that received phosphate buffered saline, *Breg* MI mice that received regulatory B cells, *Control B* MI mice that received control B cells, *LVEDD* left ventricular end-diastolic dimension, *LVESD* left ventricular end-systolic dimension, *HW/BW* heart weight/body weight ratio, *LW/BW* lung weight/body weight ratio

**Fig. 2:**
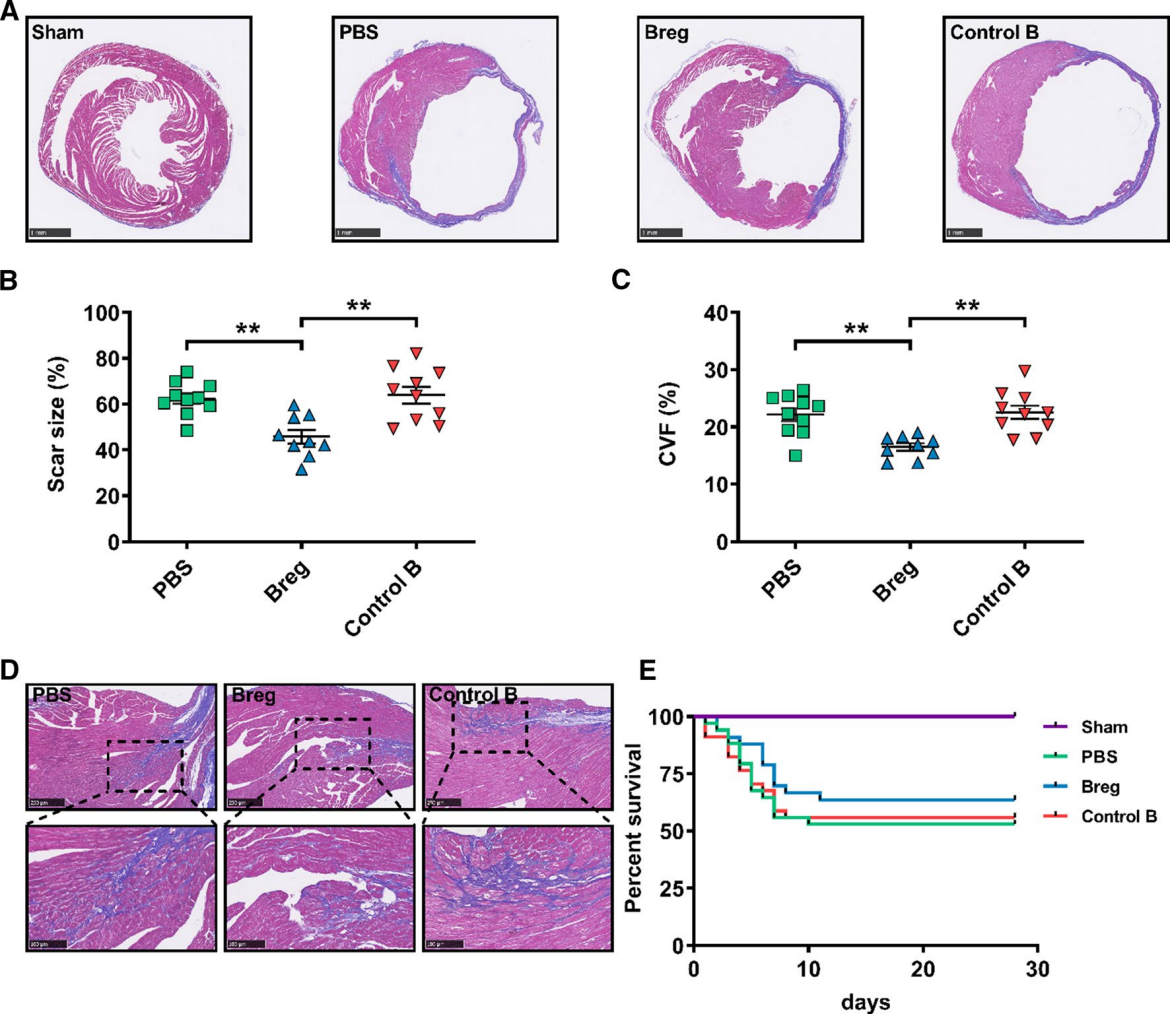
Adoptive transfer of Bregs reduces scar size and fibrosis post-MI.** a** Representative photomicrographs of scar size evaluated by Masson trichrome staining at day 28 post-MI. Scale bar: 1 mm. **b** Quantitative analysis of scar size evaluated by Masson trichrome staining at day 28 post-MI. *n* = 9–10 per group. **c** Fibrosis assessed by CVF in the peri-infarct zone was compared among the different treatments at day 28 post-MI. *n* = 9–10 per group. **d** Representative images showing collagen deposition (blue) evaluated by Masson trichrome staining 28 days after MI. Scale bar: 250 μm (top) or 100 μm (bottom). **e** Survival analysis of sham mice (*n* = 18), PBS-treated MI mice (*n* = 34), Breg-treated MI mice (n = 33), control B cell-treated mice (*n* = 34) up to 28 days following the operation. Data are expressed as means ± SEM. **P < 0.01. Data in **b** and **c** were analyzed by one-way ANOVA, followed by Tukey’s post hoc test. Survival distributions were estimated by the Kaplan–Meier method and compared by log-rank test. *Sham* sham-operated group, *PBS* MI mice that received phosphate buffered saline, *Breg* MI mice that received regulatory B cells, *Control B* MI mice that received control B cells, *CVF* collagen volume fraction

To investigate the role of exogenous Bregs in ventricular remodeling after MI, we isolated Bregs using magnetic-activated cell sorting (MACS) with purity greater than 90% and transferred them into the MI models (Supplementary Fig. 1). Cardiac function was analyzed 28 day post-MI by echocardiography. As shown in Fig. 1, Breg transfer led to increased left ventricular systolic indexes, including ejection fraction and fractional shortening, decreased left ventricular dilation indexes, including left ventricular end-diastolic dimension (LVEDD) and left ventricular end-systolic dimension (LVESD), and reduced heart and lung weight/body weight ratios, underscoring the improvement in cardiac function compared to those of phosphate buffered saline (PBS)- and control B cell-treated mice (Fig. 1). In addition, Masson trichrome staining revealed reduced scar size in mice that received Bregs (Fig. 2a, b). Consistently, interstitial fibrosis evaluated by collagen volume fraction in the peri-infarct zone was also decreased by the treatment (Fig. 2c, d). However, except for the sham group, the survival rates were not significantly different among the three groups (Fig. 2e).

In addition to MACS, fluorescence-activated cell sorting (FACS) is another commonly used method to isolate Bregs. Thus, we employed IL-10-GFP knock-in mice to verify the function of Bregs in MI. As expected, IL-10-GFP^+^ Bregs isolated by FACS also showed a protective role in MI, as demonstrated by improvement in cardiac function and reduction in scar size and interstitial fibrosis compared to those of PBS and control B cell treatment groups (Supplementary Figs. 2 and 3). Overall, these results corroborate the protective role of Bregs and indicate the therapeutic potential of Bregs in reducing cardiac remodeling after MI.

#### Breg transfer does not alter the infiltration of neutrophils or T cells into the myocardium

**Fig. 3:**
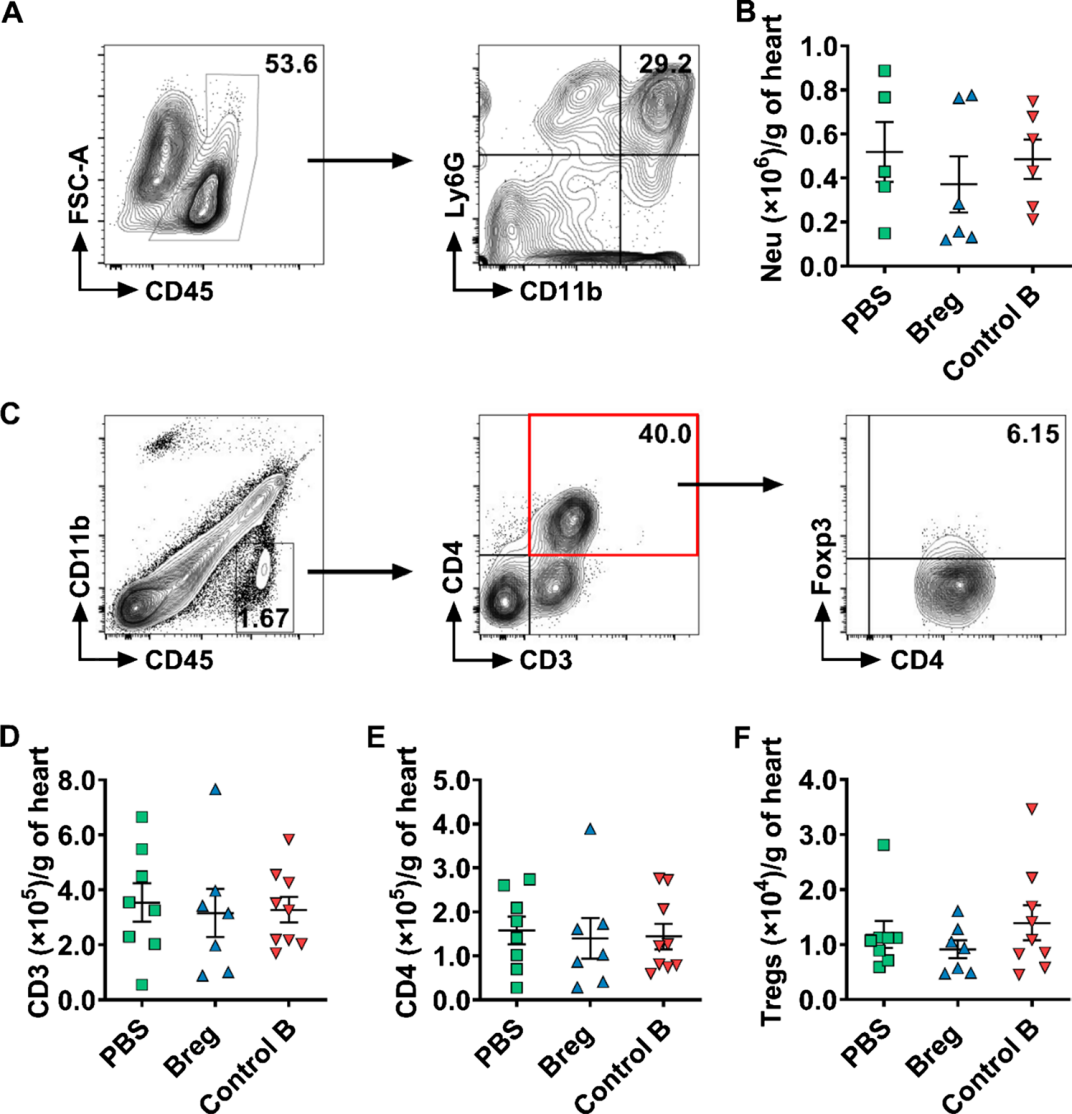
Bregs do not affect the infiltration of neutrophils or T cells into the myocardium following MI.** a** Representative flow cytometric images of neutrophils (gated on CD45^+^CD11b^+^Ly6G^+^) in the heart 3 day post-MI. **b** Absolute numbers of neutrophils infiltrating the heart were analyzed. *n* = 5–6 per group. **c** Representative flow cytometric images of CD3^+^ T cells (gated on CD45^+^CD11b^−^CD3^+^), CD4^+^ T cells (gated on CD45^+^CD11b^−^CD3^+^CD4^+^) and Tregs (gated on CD45^+^CD11b^−^CD3^+^CD4^+^Foxp3^+^) in the heart 7 days after MI. **d**–**f** Absolute numbers of CD3^+^ T cells (**d**), CD4^+^ T cells (**e**) and Tregs (**f**) infiltrating the heart were analyzed. n = 7–9 per group. Data are expressed as means ± SEM. Data in **b**, **e** and **f** were analyzed by Kruskal–Wallis test with Dunn’s multiple comparisons test. Data in **d** were analyzed by one-way ANOVA, followed by Tukey’s post hoc test. *PBS* MI mice that received phosphate buffered saline, *Breg* MI mice that received regulatory B cells, *Control B* MI mice that received control B cells, *Neu* neutrophils

Massive numbers of inflammatory cells, such as neutrophils and T cells, infiltrate into the myocardium after MI [11, 18, 51]. Bregs were reported to regulate a variety of immune cells [40, 48]. To understand the mechanisms involved in Breg-mediated beneficial effects, we assessed the infiltration of the inflammatory cells after Breg transfer. The infiltration of neutrophils in the injured myocardium peaks at day 3 after MI, while the accumulation of T cells peaks at day 7 [51]. Thus, the counts of neutrophils and T cells were detected at their respective peak time. Flow cytometric data showed that the numbers of CD11b^+^Ly6G^+^ neutrophils, CD3^+^ T cells, CD4^+^ T cells and CD4^+^Foxp3^+^ Tregs showed no significant difference among the PBS, Breg or control B cell groups (Fig. 3).

#### Breg transfer decreases the infiltration of monocytes into the myocardium

**Fig. 4:**
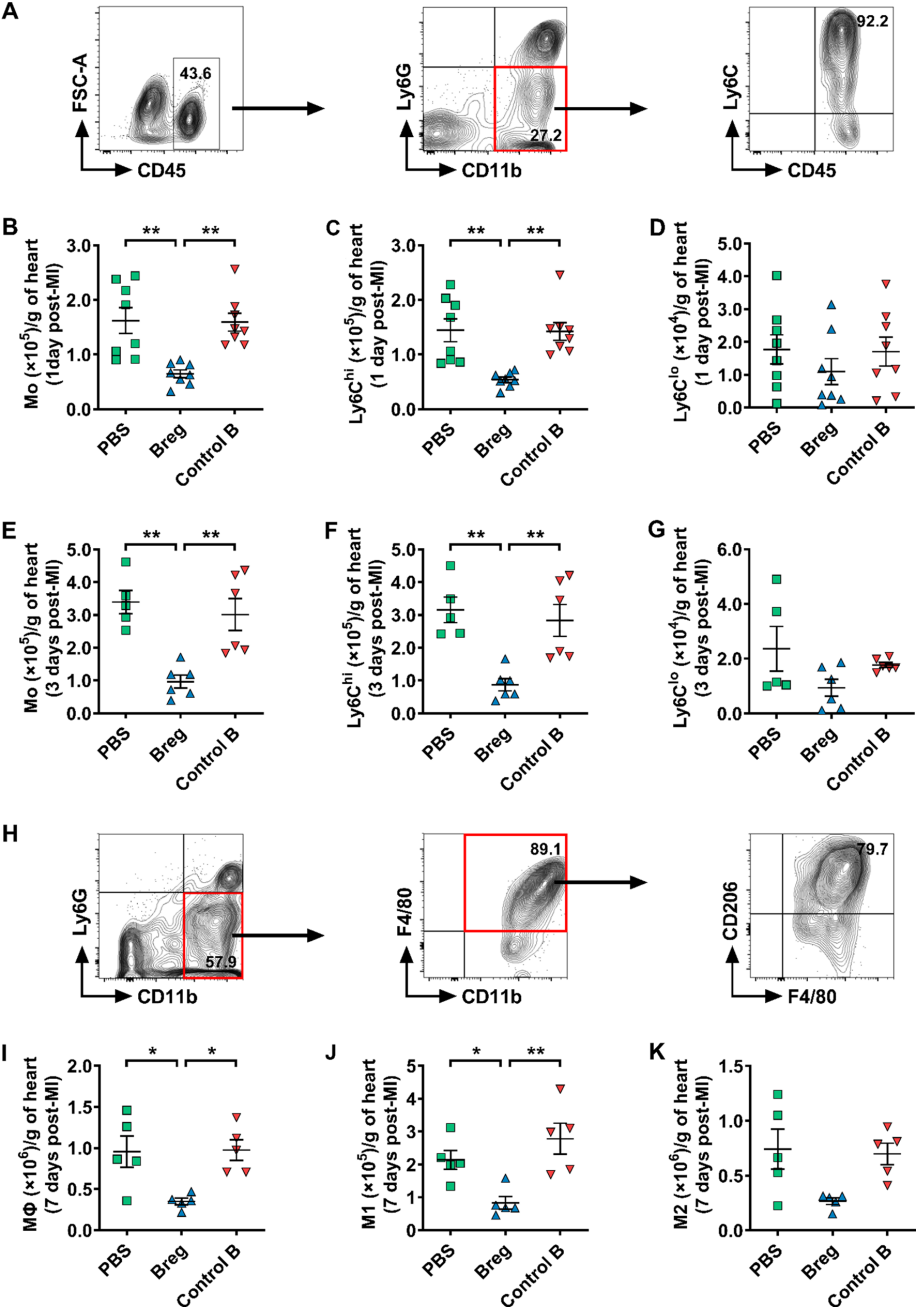
Bregs impair monocyte infiltration into the heart after MI. **a** Leukocytes were collected from the heart 1 and 3 day post-MI and stained for CD45, CD11b, Ly6G and Ly6C. Representative flow cytometric images of monocytes (gated on CD45^+^CD11b^+^Ly6G^−^) are shown. **b**–**d** Absolute numbers of monocytes and their subpopulations in the heart 1 day post-MI were analyzed. n = 8 per group. **e**–**g** Absolute numbers of monocytes and their subpopulations in the heart 3 day post-MI were analyzed. *n* = 5–6 per group. **h** Leukocytes were collected from the heart 7 day post-MI and stained for CD45, CD11b, Ly6G, F4/80 and CD206. Representative flow cytometric images of macrophages (gated on CD45^+^CD11b^+^Ly6G^−^F4/80^+^) are shown. **i**–**k** Absolute numbers of macrophages and their subpopulations in the heart at day 7 post-MI were analyzed. *n* = 5 per group. Data are expressed as means ± SEM. *P < 0.05, **P < 0.01. Data in **b**, **c**, **g**, **i** and **k** were analyzed by Kruskal–Wallis test with Dunn’s multiple comparisons test. Data in **d**–**f** and **j** were analyzed by one-way ANOVA, followed by Tukey’s post hoc test. *PBS* MI mice that received phosphate buffered saline, *Breg* MI mice that received regulatory B cells, *Control B* MI mice that received control B cells, *Mo* monocytes, *MΦ* macrophages

Previous studies reported that monocytes were key players in inflammatory expansion and wound healing post-MI [7, 27]. After MI, monocytes are quickly recruited to the infarcted heart within 24 h [43], and the number of infiltrated monocytes reaches a maximum on day 3 [53]. Accordingly, we focused on monocyte infiltration in the infarcted myocardium on day 1 and day 3 post-MI. Interestingly, Breg-transferred mice showed a reduced number of infiltrated monocytes in the myocardium compared with PBS- and control B cell-treated mice (Fig. 4b) 1 day after MI. We also assessed the subset composition of monocytes on the basis of Ly6C expression. The number of Ly6C^hi^ monocytes was significantly reduced in Breg-transferred mice, while the number of Ly6C^lo^ monocytes was not changed (Fig. 4c, d), indicating that proinflammatory Ly6C^hi^ monocytes were likely to account for the reduction of monocytes in response to Breg treatment. Furthermore, monocyte infiltration in the heart of Breg-transferred mice was also inhibited on day 3 after MI (Fig. 4e). Likewise, the number of Ly6C^hi^ but not Ly6C^lo^ monocytes was reduced at this timepoint (Fig. 4f, g). After entering into the injured heart, abundant monocytes differentiate into macrophages which reach the peak at day 7 post-MI [51]. Hence, we detected the number and subset composition of macrophages in the infarcted heart at this timepoint. Consistent with our previous results, replenishment of exogenous Bregs impaired the accumulation of macrophages especially for M1-type macrophages (Fig. 4i, j). Besides, M2-type macrophages also showed a downward trend after Breg transfusion, although there was no statistical difference among the different groups (Fig. 4k).

#### Breg transfer inhibits monocyte mobilization and recruitment

**Fig. 5:**
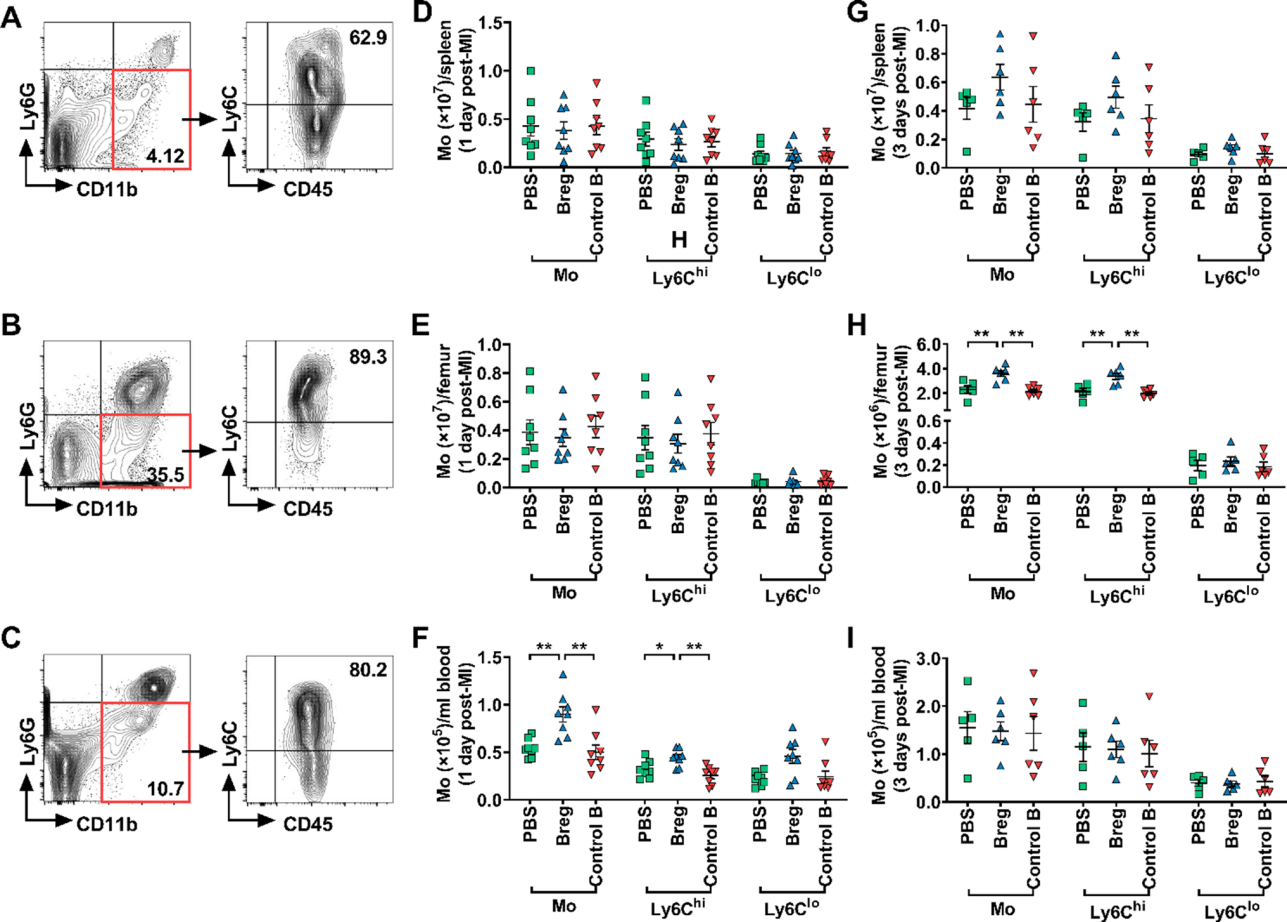
Breg transfer inhibits monocyte mobilization and recruitment. **a**–**c** Monocytes were collected from different tissues 1 and 3 days after MI and stained for CD45, CD11b, Ly6G and Ly6C. Representative flow cytometric images of monocytes are shown, respectively. **d**–**f** Absolute numbers of monocytes and their subpopulations in the spleen (**d**), bone marrow (**e**), and blood (**f**) at day 1 post-MI were analyzed. *n* = 8 per group. **g**–**i** Absolute numbers of monocytes and their subpopulations in the spleen (**g**), bone marrow (**h**), and blood (**i**) at day 3 post-MI were analyzed. *n* = 5–6 per group. Data are expressed as means ± SEM. *P < 0.05, **P < 0.01. Data in **d**, **e** and **f** were analyzed by one-way ANOVA, followed by Tukey’s post hoc test (monocytes and Ly6C^hi^ monocytes) or Kruskal–Wallis test with Dunn’s multiple comparisons test (Ly6C^lo^ monocytes). Data in **g** were analyzed by one-way ANOVA, followed by Tukey’s post hoc test (Ly6C^lo^ monocytes) or Kruskal–Wallis test with Dunn’s multiple comparisons test (monocytes and Ly6C^hi^ monocytes). Data in **h** and **i** were analyzed by one-way ANOVA, followed by Tukey’s post hoc test. *PBS* MI mice that received phosphate buffered saline, *Breg* MI mice that received regulatory B cells, *Control B* MI mice that received control B cells, *Mo* monocytes

Monocytes are produced in the bone marrow and spleen. Spleen is the main source of monocytes that are recruited to the infarcted heart within 1 day after MI. Thereafter, both bone marrow and spleen release monocytes, which enter the bloodstream and recruit to the injured myocardium [7, 43]. To explore the process of Bregs preventing monocytes from infiltrating into the myocardium, monocytes were collected from the spleen, bone marrow and peripheral blood 1 day after MI and analyzed by flow cytometry, and the results showed that the number of monocytes from Breg-treated mice increased in the blood, but not altered in the spleen or bone marrow (Fig. 5a–f). However, monocyte compartmentalization was differentially altered in Breg-treated mice on day 3 after MI, as revealed by enhanced retention in the bone marrow, but the numbers of monocytes in the blood and spleen were not changed (Fig. 5g–i). These results suggest that Bregs affect different processes of monocyte migration: Bregs inhibit monocyte recruitment to the heart from the peripheral blood with no influence on mobilization from the spleen or bone marrow on day 1, whereas monocyte mobilization from the bone marrow to the blood was impaired on day 3.

#### Breg transfer reduces CCR2 expression in monocytes

We then explored the mechanism of the regulation of Bregs on monocytes. First, we examined the possible location, where Bregs acted on monocytes. Tracing experiments were performed to determine the distribution of transferred Bregs in the recipients. Bregs derived from CD45.1 background mice were purified by MACS and then transferred to MI mice immediately after the coronary artery was ligated. We examined the presence of transferred cells in the heart, peripheral blood, spleen and bone marrow 1, 3, 7 and 14 days after MI. Flow cytometric data showed that transferred Bregs mainly accumulated in the bone marrow and spleen, while a small proportion was detected in the heart and peripheral blood, suggesting that Bregs may act on monocytes in the bone marrow and spleen (Supplementary Fig. 4).

**Fig. 6:**
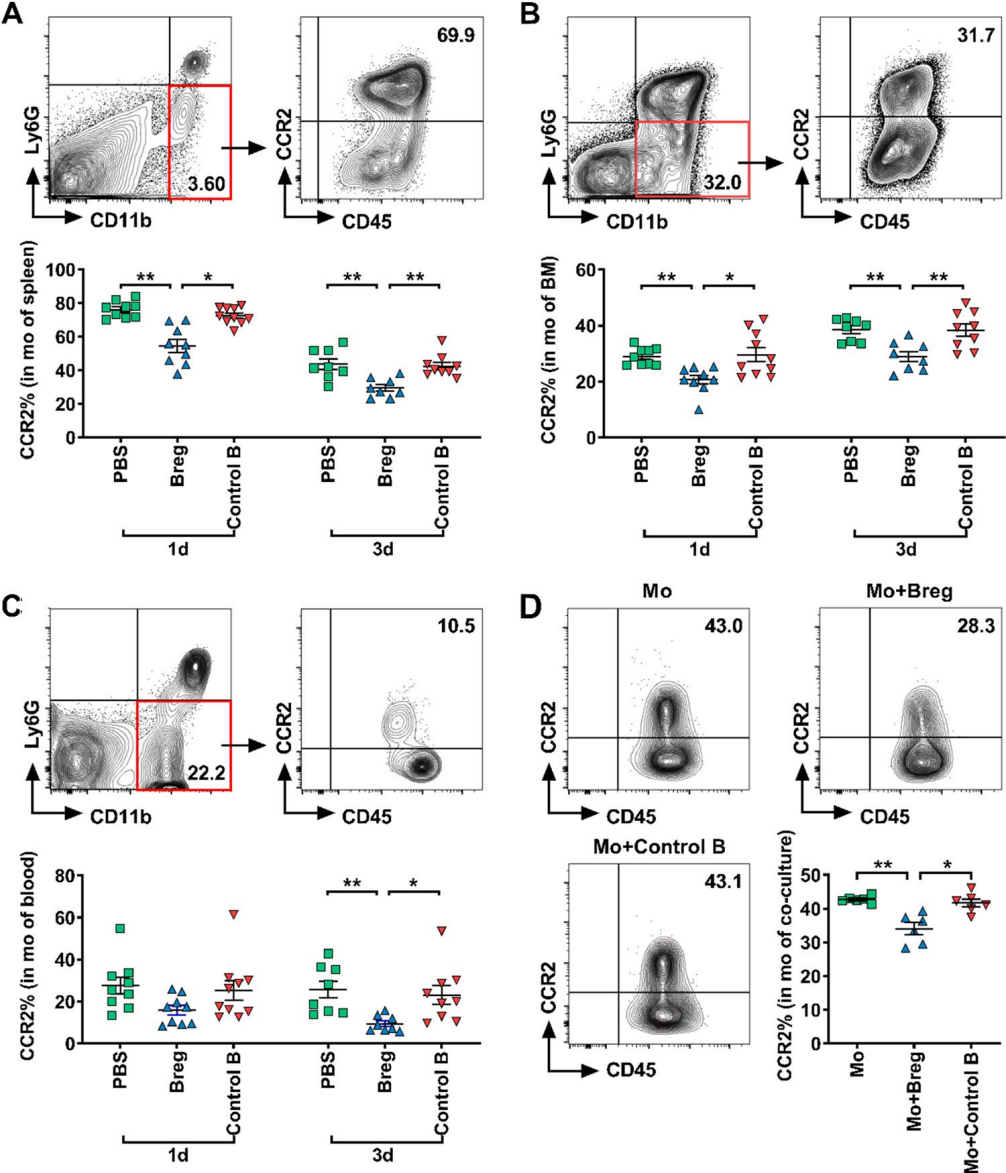
Bregs reduce CCR2 expression in monocytes. **a**–**c** Monocytes were collected from different tissues 1 and 3 days after MI and stained for CD45, CD11b, Ly6G and CCR2. Representative flow cytometric images and analysis of CCR2 expression in monocytes after MI in the spleen (**a**), bone marrow (**b**) and blood (**c**) are shown. *n* = 8–10 per group. **d** Monocytes sorted from the spleen were co-cultured with Bregs for 1 day, and the CCR2 expression in monocytes were detected using flow cytometry. Representative flow cytometric images and analysis of CCR2 expression in monocytes among the different treatments are shown. *n* = 6 per group. Data are expressed as means ± SEM. *P < 0.05, **P < 0.01. Data in **a** and **b** were analyzed by one-way ANOVA, followed by Tukey’s post hoc test (3d) or Kruskal–Wallis test with Dunn’s multiple comparisons test (1d). Data in **c** and **d** were analyzed by Kruskal–Wallis test with Dunn’s multiple comparisons test. *PBS* MI mice that received phosphate buffered saline, *Breg* MI mice that received regulatory B cells, *Control B* MI mice that received control B cells, *1d* 1 day post-MI, *3d* 3 day post-MI, *Mo* monocytes cultured alone, *Mo + Breg* monocytes co-cultured with regulatory B cells, *Mo + Control B* monocytes co-cultured with control B cells, *mo* monocytes, *BM* bone marrow

Next, we examined which molecule was responsible for the effect of Bregs. After MI, monocytes are mobilized from the spleen and bone marrow into the peripheral blood through the angiotensin II type 1 receptor (AT1R)- and CCR2-dependent pathways, respectively, and then recruited from the peripheral blood to the heart through the CCR2-mediated mechanism [7, 15, 35, 43]. Chemokines C–C motif chemokine ligand 2 (CCL2) and C–C motif chemokine ligand 7 (CCL7) as the two major ligands of CCR2 can interact with the latter to cause monocyte migration. To explore which molecule was affected by Bregs, we first examined the potential change in AT1R expression on splenic monocytes, but found no change in mice that were treated with Breg transfer (Supplementary Fig. 5a), which was in accordance with the result that monocyte number in the spleen was not altered, as monocyte mobilization from the spleen was proved to be associated with AT1R rather than CCR2. Then, we focused on CCR2 and its two major ligands, CCL2 and CCL7. We found that these two chemokines in serum were not significantly affected by Breg transfer either on day 1 or day 3 after MI (Supplementary Fig. 6), indicating that they were not targets of Breg treatment. Next, we measured CCR2 expression in monocytes. On day 1 after MI, Breg transfer led to reduced CCR2 expression in monocytes from the spleen and bone marrow with a mild but insignificant reduction in monocytes in the blood (Fig. 6a–c). Similarly, on day 3 after MI, monocytes from the spleen, blood and bone marrow all showed reduced CCR2 expression in Breg-transferred mice (Fig. 6a–c). Besides, we further verified the decreased CCR2 expression in splenic monocytes after Breg transfer by RT-qPCR (Supplementary Fig. 5b). Thus, our results indicate that Bregs down-regulate the expression of CCR2 in monocytes, thereby reducing the mobilization of monocytes from the bone marrow to the blood and recruitment from the blood to the myocardium.

To directly verify the action of Bregs on monocytes, we also tested the effect of Bregs on CCR2 expression in monocytes in vitro. Under stimulation with lipopolysaccharide, monocytes were co-cultured with Bregs or control B cells for 24 h. Monocyte CCR2 expression was significantly reduced in the presence of Bregs compared with that of cells cultured with medium or control B cells, as detected by flow cytometry (Fig. 6d).

#### IL-10 plays a critical role in Breg-mediated protection against MI

**Fig. 7:**
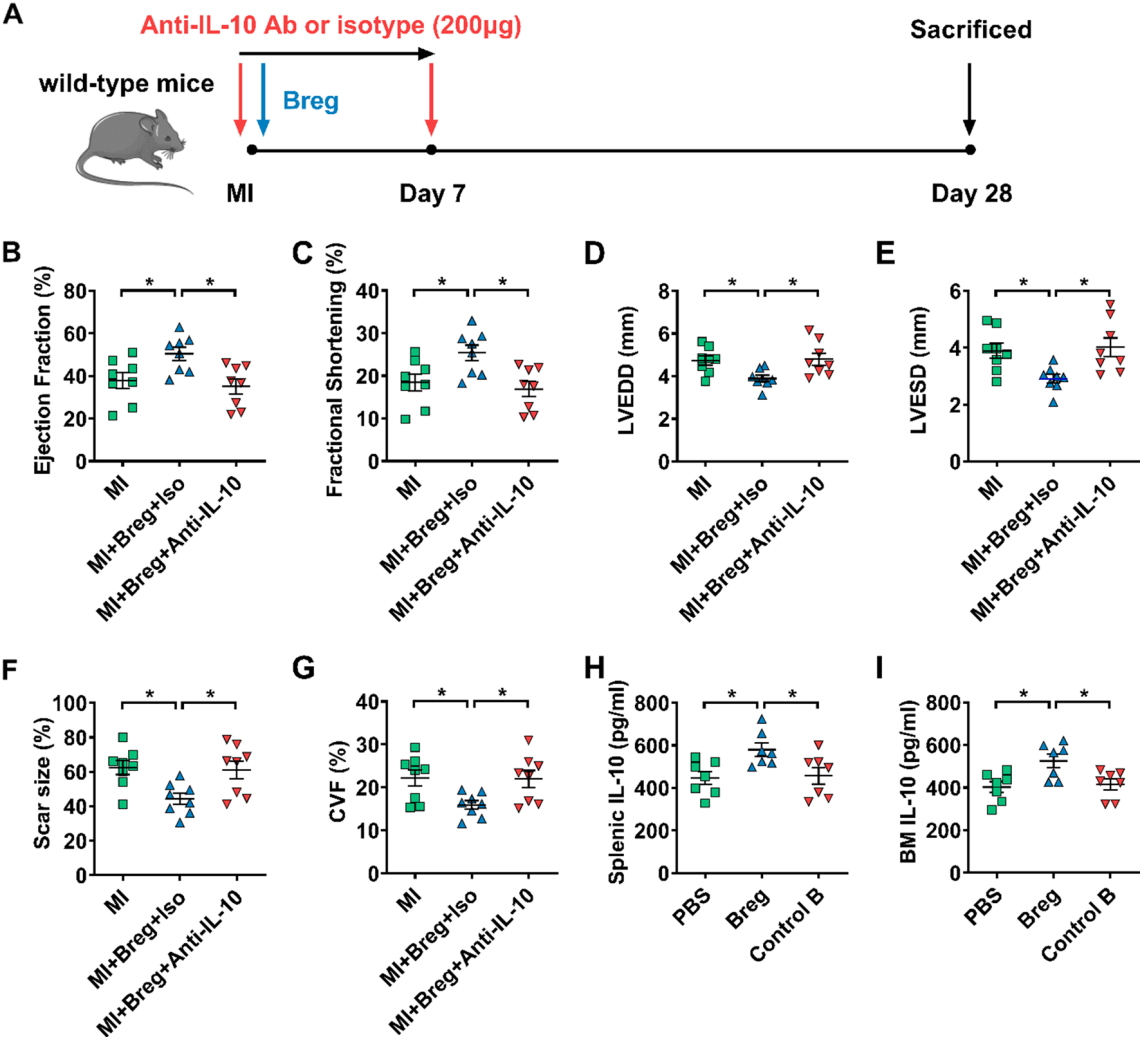
IL-10 plays a critical role in Breg-mediated protection against MI. **a** Experimental procedures and timeline of surgery and treatment are shown. MI mice were administered with Bregs along with the anti-IL-10 antibody or isotype control antibody. **b**–**e** Ejection fraction (**b**), fractional shortening (**c**), LVEDD (**d**) and LVESD (**e**) were assessed using echocardiography 28 day post-MI. *n* = 8 per group. **f**, **g** Scar size (**f**) and CVF (**g**) were measured by Masson trichrome staining 28 day post-MI. n = 8 per group. **h**, **i** PBS, Bregs or control B cells were injected intravenously after the coronary artery was ligated and the expression of IL-10 in the spleen (**h**) and bone marrow (**i**) was measured by ELISA at day 1 post-MI. *n* = 7 per group. Data are expressed as means ± SEM. *P < 0.05. Data in **b**–**i** were analyzed by one-way ANOVA, followed by Tukey’s post hoc test. *MI* MI mice control group, *MI + Breg + Iso* MI mice that received regulatory B cells along with the isotype control antibody, *MI + Breg + Anti-IL-10* MI mice that received regulatory B cells along with the anti-IL-10 antibody, *PBS* MI mice that received phosphate buffered saline, *Breg* MI mice that received regulatory B cells, *Control B* MI mice that received control B cells, *Ab* antibody, *LVEDD* left ventricular end-diastolic dimension, *LVESD* left ventricular end-systolic dimension, *CVF* collagen volume fraction, *BM* bone marrow

IL-10, TGF-β1 and IL-35 contribute to Breg functions in various experimental systems [28, 32, 36, 39, 40, 42, 45, 47, 52]. We also verified the preferential expression of these cytokines by Bregs in our pilot study (Supplementary Fig. 7). To examine the potential roles of these molecules in the MI model, we transferred Bregs in the presence of anti-IL-10, anti-TGF-β1, anti-EBI3 or isotype control antibodies, and then compared the indexes of cardiac function and ventricular remodeling. Transfer of Bregs with the isotype control antibody improved cardiac function and ameliorated ventricular remodeling, as shown by decreased LVEDD and LVESD, enhanced ejection fraction and fractional shortening, reduced scar size and collagen volume fraction. These effects were abrogated after the transfer of Bregs with the anti-IL-10 antibody (Fig. 7b–g). Moreover, to provide direct evidence of IL-10 production of Bregs in vivo, we detected the levels of IL-10 at day 1 after MI in the bone marrow and spleen, where transferred Bregs mainly accumulated. The results showed that Bregs induced increased IL-10 expression in both two tissues (Fig. 7h, i). However, transfer of Bregs in the presence of anti-TGF-β1 and anti-EBI3 antibodies still had protective effects on MI, with comparable cardiac function, scar size and fibrosis compared to the effects of the transfer of Bregs with isotype control antibodies (Supplementary Figs. 8 and 9). These results suggest that IL-10 but not TGF-β1 or IL-35 is essential for mediating Breg-induced protection against MI.

**Fig. 8:**
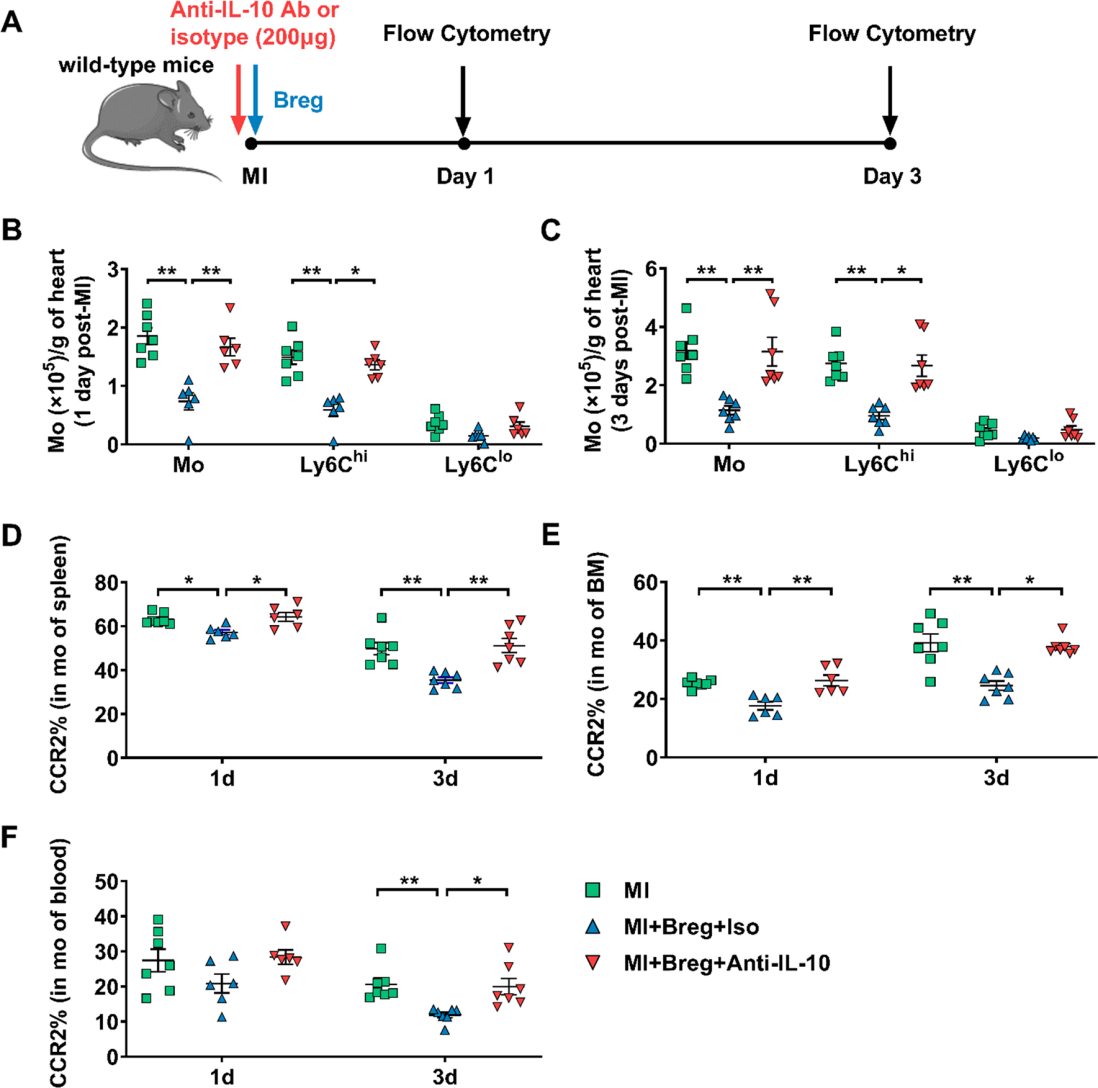
Anti-IL-10 antibody antagonizes the effect of Bregs on monocytes in vivo.** a** Experimental procedures and timeline of surgery and treatment are shown. MI mice were administered with Bregs along with the anti-IL-10 antibody or isotype control antibody. **b, c** Numbers of monocytes and their subsets that infiltrated into the heart at day 1 (**b**) and day 3 (**c**) after MI were detected using flow cytometry. *n* = 6–7 per group. **f** CCR2 expression in monocytes from the spleen (**d**), bone marrow (**e**), and blood (**f**) was measured 1 and 3 day post-MI. *n* = 6–7 per group. Data are expressed as means ± SEM. *P < 0.05, **P < 0.01. Data in **b** were analyzed by one-way ANOVA, followed by Tukey’s post hoc test (monocytes and Ly6C^lo^ monocytes) or Kruskal–Wallis test with Dunn’s multiple comparisons test (Ly6C^hi^ monocytes). Data in **c** were analyzed by Kruskal–Wallis test with Dunn’s multiple comparisons test. Data in **d** were analyzed by one-way ANOVA, followed by Tukey’s post hoc test (3d) or Kruskal–Wallis test with Dunn’s multiple comparisons test (1d). Data in **e** and **f** were analyzed by one-way ANOVA, followed by Tukey’s post hoc test (1d) or Kruskal–Wallis test with Dunn’s multiple comparisons test (3d). *MI* MI mice control group, *MI + Breg + Iso* MI mice received regulatory B cells along with the isotype control antibody, *MI + Breg + Anti-IL-10* MI mice received regulatory B cells along with the anti-IL-10 antibody, *Ab* antibody, *Mo or mo* monocytes, *BM* bone marrow

**Fig. 9:**
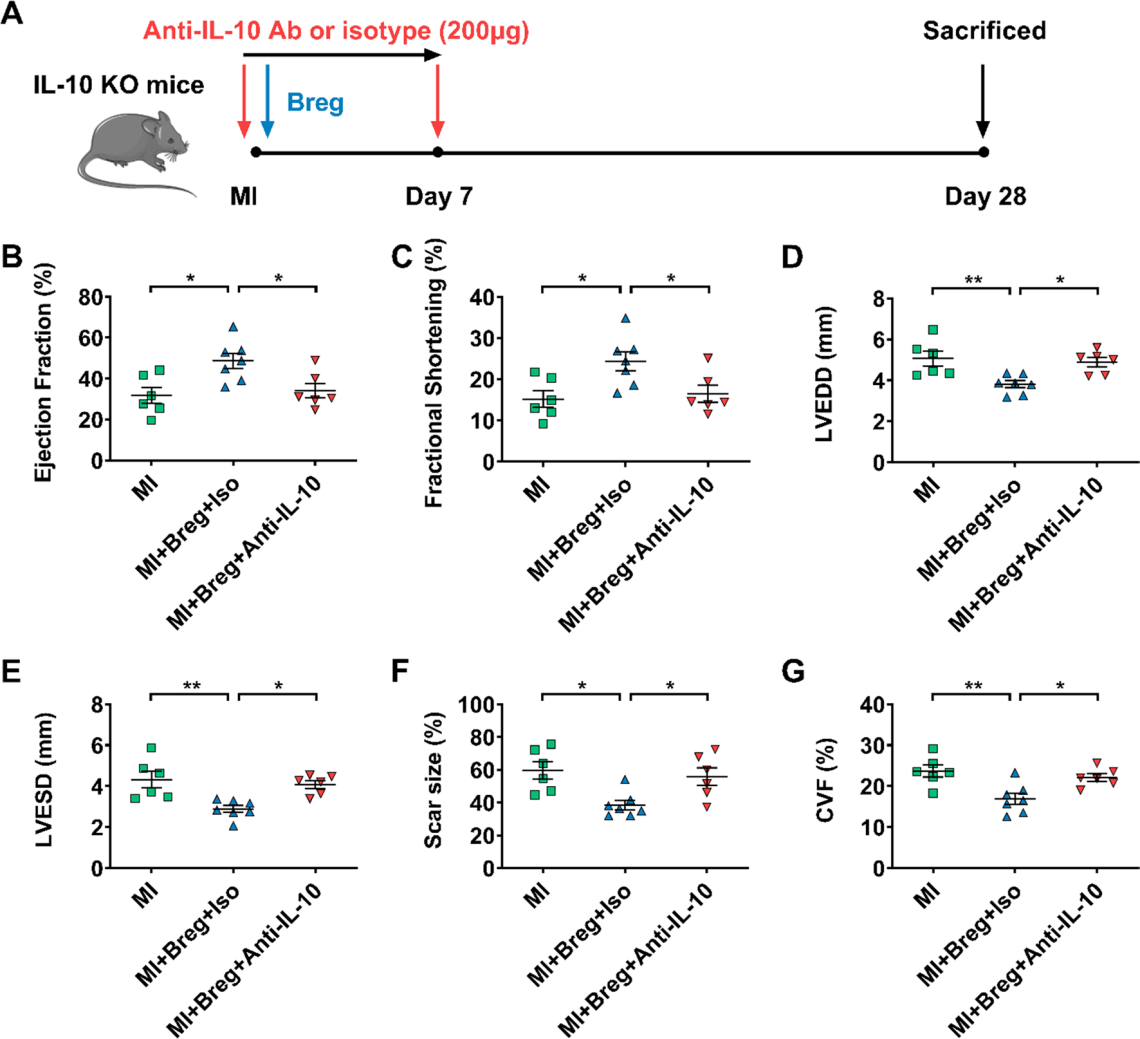
Bregs protect against MI through an IL-10-dependent mechanism. **a** Experimental procedures and timeline of surgery and treatment are shown. IL-10 KO MI mice were administered with Bregs along with the anti-IL-10 antibody or isotype control antibody. **b**–**e** Ejection fraction (**b**), fractional shortening (**c**), LVEDD (**d**) and LVESD (**e**) were assessed using echocardiography 28 days after MI. *n* = 6–7 per group. **f**, **g** Scar size (**f**) and CVF (**g**) were measured by Masson trichrome staining 28 days after MI. *n* = 6–7 per group. Data are expressed as means ± SEM. *P < 0.05, **P < 0.01. Data in **b**–**g** were analyzed by one-way ANOVA, followed by Tukey’s post hoc test. *MI* MI mice control group, *MI + Breg + Iso* MI mice that received regulatory B cells along with the isotype control antibody, *MI + Breg + Anti-IL-10* MI mice that received regulatory B cells along with the anti-IL-10 antibody, *KO* knock-out, *Ab* antibody, *LVEDD* left ventricular end-diastolic dimension, *LVESD* left ventricular end-systolic dimension, *CVF* collagen volume fraction

**Fig. 10:**
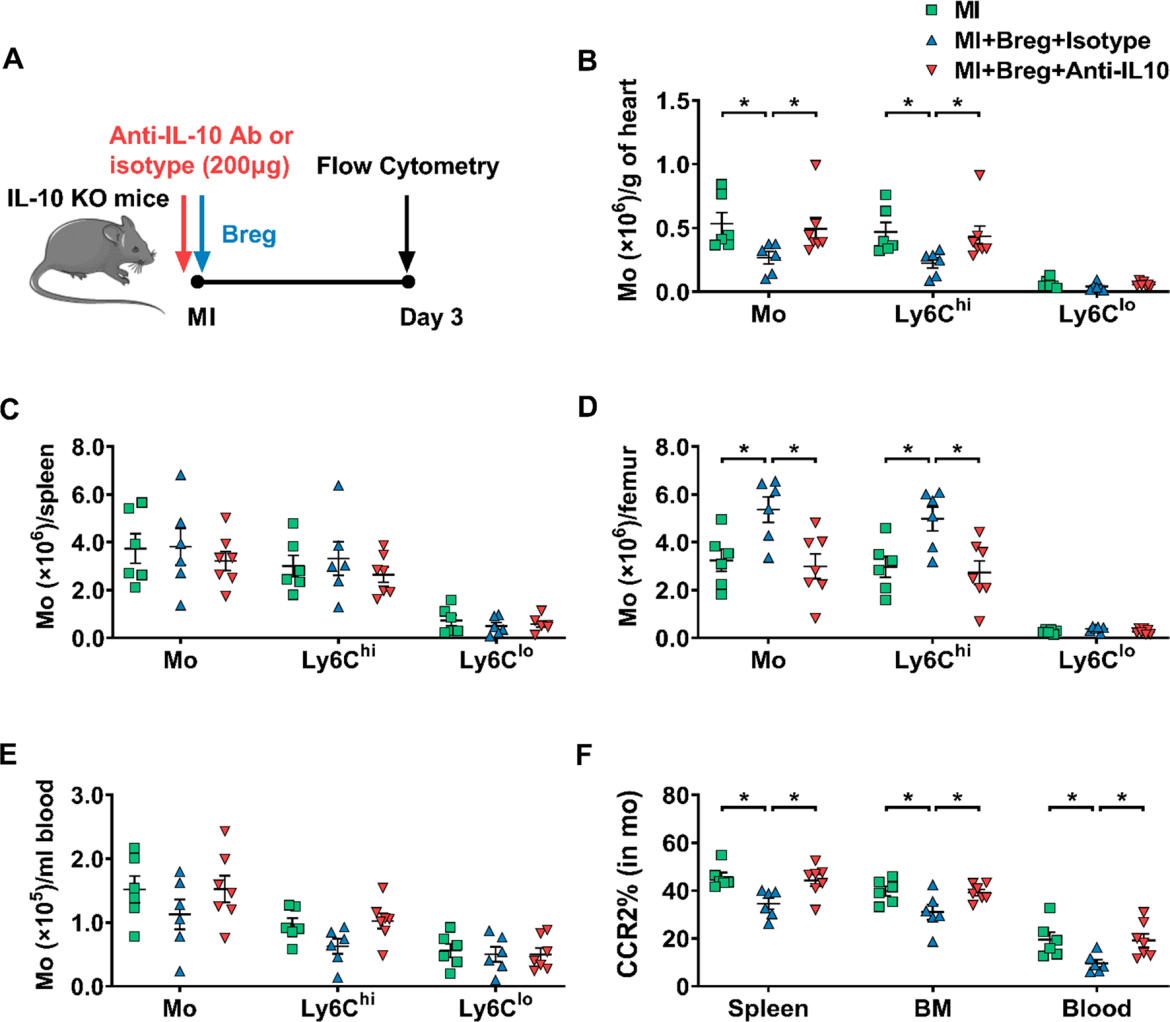
Anti-IL-10 antibody antagonizes the effect of Bregs on monocytes in vivo. **a** Experimental procedures and timeline of surgery and treatment are shown. IL-10 KO MI mice were administered with Bregs along with the anti-IL-10 antibody or isotype control antibody. **b**–**e** Numbers of monocytes and their subsets in the heart (**b**), spleen (**c**), bone marrow (**d**), and blood (**e**) at day 3 after MI were detected using flow cytometry. n = 6–7 per group. **f** CCR2 expression in monocytes from the spleen, bone marrow and blood were measured 3 day post-MI. *n* = 6–7 per group. Data are expressed as means ± SEM. *P < 0.05. Data in **b** were analyzed by one-way ANOVA, followed by Tukey’s post hoc test (Ly6C^lo^ monocytes) or Kruskal–Wallis test with Dunn’s multiple comparisons test (monocytes and Ly6C^hi^ monocytes). Data in **c**,** d** and **e** were analyzed by one-way ANOVA, followed by Tukey’s post hoc test. Data in **f** were analyzed by one-way ANOVA, followed by Tukey’s post hoc test (BM and blood) or Kruskal–Wallis test with Dunn’s multiple comparisons test (spleen). *MI* MI mice control group, *MI + Breg + Iso* MI mice received regulatory B cells along with the isotype control antibody, *MI + Breg + Anti-IL-10* MI mice received regulatory B cells along with the anti-IL-10 antibody, *KO* knock-out, *Ab* antibody, *Mo or mo* monocytes, *BM* bone marrow

Next, we investigated whether IL-10 was involved in Breg-mediated regulation of monocytes. Myocardial infiltration of monocytes was evaluated 1 or 3 day post-MI. While Breg transfer with the isotype control antibody suppressed the recruitment of monocytes, specifically Ly6C^hi^ monocytes, in the myocardium, the IL-10 antibody abrogated the suppressive effect of Bregs (Fig. 8b, c). Moreover, the IL-10 antibody also inhibited Breg-mediated downregulation of monocyte CCR2 expression (Fig. 8d–f). It has been shown that various subsets of immune cells could produce IL-10, such as macrophages, dendritic cells, T cells and natural killer cells [13, 33, 37]. Therefore, to further confirm Bregs exert their protective effects via the production of IL-10, we adoptively transferred Bregs into IL-10 KO MI mice. Compared with IL-10 KO MI mice, IL-10 KO MI mice which were administered with Bregs improved the cardiac function, decreased the scar size and attenuated the interstitial fibrosis of the peri-infarct area. In addition, adoptive transfer of Bregs to IL-10 KO mice also reduced the infiltration of monocytes and their expression of CCR2 after MI. However, consistent with our previous results, the protective effects of transferred Bregs in IL-10 KO mice disappeared after injection of anti-IL-10 antibody, indicating the critical role of IL-10 in the Breg-mediated effect on MI (Figs. 9, 10). In addition, in vitro experiments were performed to directly assess the contribution of IL-10 to the effect of Bregs on CCR2 expression. First, transwell experiments showed that disruption of cell–cell contact did not reverse the downregulation, supporting that a secretory mechanism might be responsible for the process (Supplementary Fig. 10a). Then, the anti-IL-10 antibody was added to the co-culture system of Bregs and monocytes, and the results showed that blocking IL-10 attenuated Breg-mediated downregulation of monocyte CCR2 expression (Supplementary Fig. 10b). Taken together, these data support the critical role of IL-10 in the Breg-mediated effect on monocytes.

## Discussion

In the present study, we first demonstrated a beneficial effect of exogenous Bregs on MI mice, as confirmed by improved cardiac function, decreased scar size and attenuated interstitial fibrosis. Furthermore, we revealed that the infiltration of proinflammatory monocytes was impaired after MI in Breg-transferred mice. Mechanistically, the protection was mediated by downregulating the CCR2 expression of proinflammatory monocytes. Finally, the characteristic cytokine IL-10 was proved to be critical for Breg-induced cardiac protection.

Zouggari et al. suggested that B cells accelerated MI damage by secreting CCL7 to promote the mobilization of monocytes [53]. However, the study by Goodchild et al. reported that intra-myocardial injection of bone-marrow-derived B cells improved cardiac function after MI [14]. The contradictory roles of B cells in MI can be explained by the fact that B cells are heterogeneous. Thus, the precise B cell subset involved in this setting needs to be examined in more detail. In this context, a recent study reported that B cell-specific deletion of IL-10 worsened cardiac function and exacerbated myocardial injury following acute MI [49]. This result suggests that Bregs might be a potential therapeutic target for MI. As Bregs were scarce in mice, we tried to explore whether exogenous Bregs could play a role in MI. IL-10^+^ Bregs, which have been studied most widely, could be differentiated from B cells at any development stages upon stimulation. They have diverse phenotypes and have been reported to be most abundant in the spleen [3, 30, 40]. To maximize the efficiency of Bregs, we directly collected B cells with IL-10 secretion potential from the spleen as exogenous supplements. Consistent with previous results [4], we found that IL-10^+^ Bregs were rare in freshly isolated B cells (data not shown), thus lipopolysaccharide was used to stimulate the isolated B cells to obtain more Bregs, and the B cells remained IL-10 negative were used as control B cells. Our results showed that transfusion of in vitro-expanded Bregs reduced myocardial scar size and myocardial fibrosis, lessened heart enlargement and improved cardiac function. These findings not only confirm the protective effect of Bregs on MI with gain-of-function experiments, but also, more importantly, suggest a potential cell-based therapy for MI.

Healing of MI requires monocytes, which contribute to inflammation, proteolysis, phagocytosis, angiogenesis, and collagen deposition [7, 15, 19, 35]. Monocytes are divided into two subsets based on varied levels of Ly6C, including Ly6C^hi^ inflammatory monocytes and Ly6C^lo^ repairing monocytes. Ly6C^hi^ monocytes remove necrotic tissues by endocytosis and, more importantly, secrete proteolytic enzymes and a large number of proinflammatory cytokines in the infarcted area, which are called inflammatory monocytes. Conversely, Ly6C^lo^ monocytes facilitate repair of the myocardium by promoting the aggregation of myofibroblasts, collagen deposition and angiogenesis [7, 35]. Our results showed that adoptive transfer of Bregs led to a decrease in the number of cardiac monocytes 1 day and 3 days after MI. Specifically, Ly6C^hi^ inflammatory monocytes, instead of Ly6C^lo^ monocytes, were reduced by Bregs. These findings support the notion that Bregs mediate cardiac protection via suppressing Ly6C^hi^ monocytes that are considered to be harmful in MI. Consistent with our results, adoptive transfer of IL-10^+^ B cells significantly reduced the circulating inflammatory monocytes during atherosclerosis development in Ldlr^−/−^ mice [6]. Interestingly, Wu et al. also showed that Bregs impacted the balance between pro- and anti-inflammatory monocytes after acute MI [49]. It is established that monocytes extravasate into the heart when a myocardial ischemic injury occurs, and most of them differentiate into macrophages subsequently. Among them, Ly6C^hi^ monocytes mainly differentiate into M1-type macrophages, while Ly6C^lo^ monocytes differentiate into M2-type macrophages [34]. Since Bregs modulate the early mobilization and infiltration of monocytes, it is presumably that Bregs also affect macrophages. As expected, our results showed that replenishment of exogenous Bregs impaired the accumulation of macrophages especially for the M1-type macrophages. M2-type macrophages also displayed a downward trend in the Breg group, although there was no statistical difference among the three groups. This may be explained by a conversion of M1- into M2-type macrophages [8, 9].

While previous study [49] did not explore the underlying mechanism of how Bregs acted on monocytes, in this study, we took deep insight to unveil it, which was beneficial to comprehensively delineate the role of Bregs in MI. First, we examined how Bregs affected monocyte migration. Within 24 h of MI, spleen is the main source of monocytes. Thereafter, both bone marrow and spleen continuously release monocytes into the blood which are subsequently recruited to the infarcted heart [7, 43]. However, the mechanisms by which monocytes mobilize from these two tissues are different. Sympathetic nerve excitation in mice after MI causes the activation of the renin–angiotensin–aldosterone system and then acts on AT1R on the surface of monocytes in the spleen, which leads to the discharge of monocytes from the spleen into the blood [43]. In contrast, the mobilization of monocytes from the bone marrow to the blood and the recruitment from the blood to the heart are mainly dependent on the CCR2 pathway [7, 15]. Therefore, to explore the process by which Bregs affect monocyte recruitment, we examined the changes of monocytes in each tissue along the migratory trajectory after MI. We first found that on day 1 after MI, the number of monocytes did not significantly change in the spleen in the Breg group compared to that of the control group, but it increased in the peripheral blood. Thus, we speculated that Bregs did not affect the mobilization of splenic monocytes but inhibited the recruitment of monocytes from the blood into the heart. Interestingly, we noticed that the number of monocytes in the bone marrow was unchanged, whereas CCR2 expression decreased in Breg-transferred mice 1 day after MI. This could presumably be due to the massive release of monocytes into the blood by the spleen instead of the bone marrow 1 day after MI. Within the first 24 h after MI, approximately half of the monocytes recruited to the ischemic myocardium are derived from the splenic reservoir [43]. However, monocyte compartmentalization was differentially altered by Breg transfer on day 3 after MI, as revealed by enhanced accumulation in the bone marrow, but unchanged in the peripheral blood and spleen. This suggests that Bregs also inhibit monocyte mobilization from the bone marrow. Notably, the number of monocytes in the peripheral blood did not change despite impaired mobilization from the bone marrow at this time, maybe because the number of monocytes released into the blood by the bone marrow and the number of blood monocytes infiltrating into the heart decreased simultaneously.

Second, we examined, where Bregs acted on monocytes. In a murine experimental stroke, transferred Bregs were mainly detected in the peripheral blood and immune organs but not in the ischemic or nonischemic brains, indicating a peripheral function of transferred Bregs [38]. However, in an allergic asthma model, injected Bregs were shown to migrate into the lung and restraint Th2- and Th17-driven inflammation locally [2]. In the present study, a tracing experiment clearly showed the process of Bregs regulating monocytes. The transferred Bregs were mainly distributed in the spleen and bone marrow after MI, suggesting that Bregs acted on monocytes mainly in these two tissues to affect their recruitment, which was consistent with the perspective that the spleen, as an immune organ, was involved in the repair and remodeling after MI [16, 29]. In contrast, supplemental Bregs were barely found in the heart either on day 1 or day 3 after MI, the timepoints at which Bregs inhibited the migration of monocytes, excluding the possibility of the heart as the location, where the transferred Bregs worked. Although endogenous Bregs were shown increased in the myocardium at day 7 after MI [49], few transferred Bregs were detected on day 7 or day 14 in our subsequent experiments, indicating different ways by which endogenous and exogenous Bregs worked in MI.

Third, the molecular mechanism responsible for Breg regulation on monocyte recruitment was examined. Bregs did not affect the expression of AT1R in the splenic monocytes or the levels of CCL2 and CCL7 in the peripheral blood; therefore, we focused on the CCR2 molecule which impacts monocyte mobilization from the bone marrow and infiltration into the heart. Transferred Bregs were found to reduce monocyte CCR2 expression in the spleen, bone marrow, and peripheral blood which was also confirmed in co-culture systems. Consistent with our results, Majmudar et al. applied monocyte-directed CCR2 siRNA in vivo and found a reduction in the number of classical monocytes at the sites of inflammation, without difference in the nonclassical monocyte subset. The authors concluded that the CCR2 siRNA could be useful as a therapeutic tool to reduce left ventricular remodeling and improve post-MI heart failure in animals [31]. Besides, another research showed that global deletion of CCR2 in mice that underwent MI also resulted in improved left ventricular remodeling [22]. The established potential of treatment targeting CCR2 makes it promising to develop Breg-based therapeutic strategies.

Different cytokines have been identified as suppressive cytokines produced by Bregs, and most studies have demonstrated a key role for B cell-derived IL-10, a hallmark cytokine of Bregs, in the suppression of different disease models, such as arthritis, experimental autoimmune encephalomyelitis and colitis [23, 32, 52]. Interestingly, IL-10, as an anti-inflammatory cytokine, has substantially been reported to promote post-MI repair and attenuate ventricular remodeling, which was related to an impaired infiltration of inflammatory cells including monocytes [21, 25]. Our study found that Bregs induced an increase of IL-10 expression in the spleen and bone marrow, presenting a prerequisite for IL-10 as a functional mediator. Moreover, we provided direct evidence that IL-10 but not TGF-β1 or IL-35 played a protective role in exogenous Breg-mediated effects on MI, which was in accordance with the results of endogenous Bregs, as B cell-specific deletion of IL-10 worsened cardiac function and exacerbated myocardial injury after MI [49]. Furthermore, we demonstrated that IL-10 was responsible for Breg-induced inhibition of monocyte recruitment to the myocardium. A previous study showed that IL-10 regulated peripheral monocyte percentages through CCR2 in Trypanosoma brucei infected mice [1]. Consistently, we provided both in vivo and in vitro evidence that IL-10 was essential for Breg-induced inhibition of CCR2 expression.

**Fig. 11:**
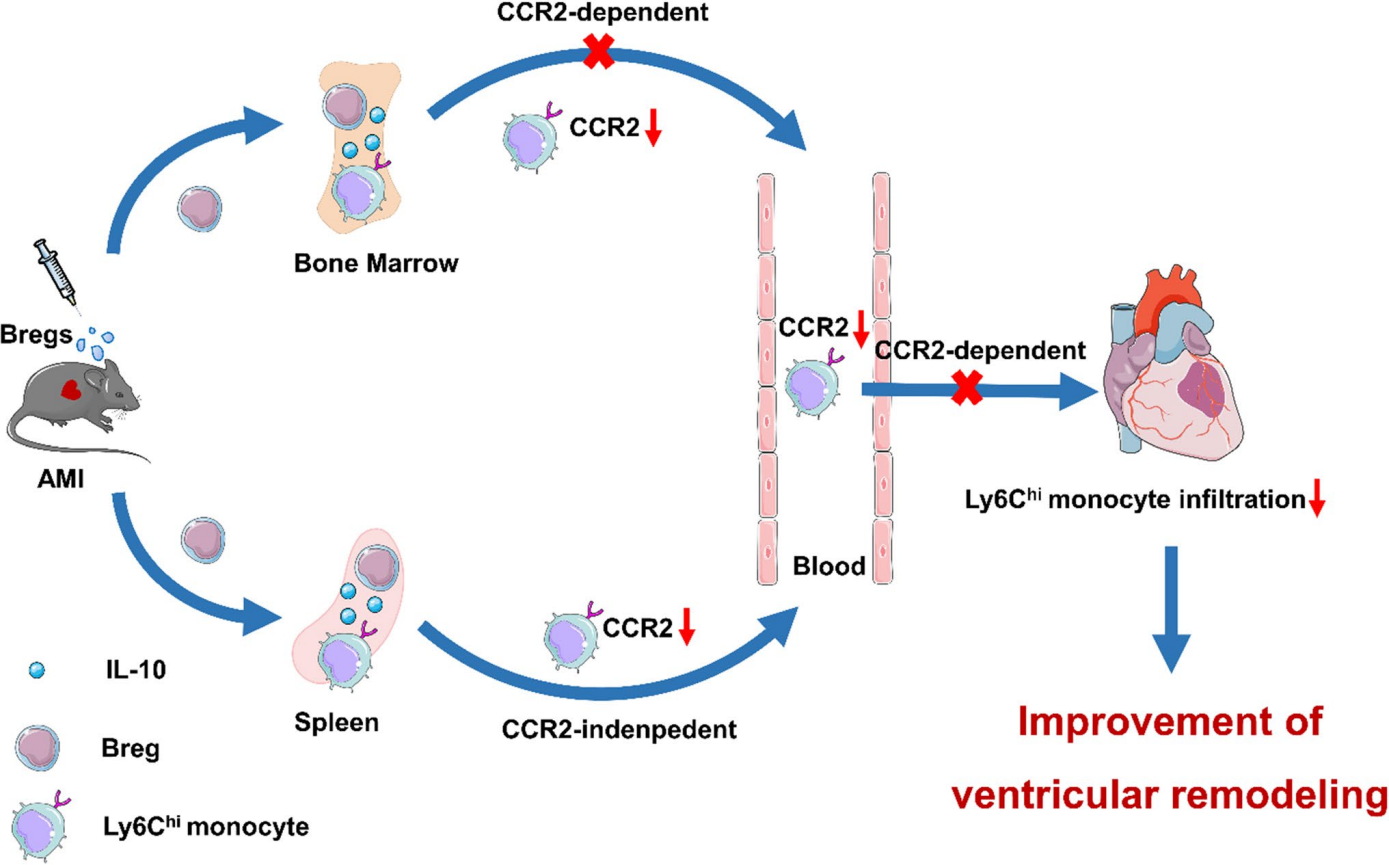
Bregs ameliorate ventricular remodeling after MI by modulating monocyte migration. Bregs adoptively transferred to MI mice migrated to the bone marrow and spleen, where they acted on monocytes by secreting IL-10 to reduce their CCR2 expression. The decline in CCR2 expression not only led to a decrease of Ly6C^hi^ monocyte recruitment from the blood to the heart, but also impaired Ly6C^hi^ monocyte mobilization from the bone marrow after MI. Thus, ventricular remodeling post-MI was ameliorated due to the reduced monocyte infiltration. *AMI* acute myocardial infarction

In summary, our study demonstrates that adoptive transfer of Bregs is protective against MI. Supplementation with exogenous Bregs exerts beneficial effects through inhibiting the expression of CCR2 in monocytes and subsequently reducing the infiltration of monocytes into the myocardium, thus facilitating cardiac healing and limiting ventricular dysfunction. Besides, IL-10 plays an indispensable role in these processes (Fig. 11). The present study provides a theoretical basis for cell therapy strategies using Bregs and provides more possibilities for the clinical treatment of MI.

### Study limitation

A major limitation of this study is that only male mice were used. Whether exogenous Bregs can ameliorate post-MI ventricular remodeling as well in female mice remains to be evaluated in further studies.

## Supplementary Information

Below is the link to the electronic supplementary material.

Supplementary file1 (DOCX 4158 KB)

## Data Availability

The data underlying this article will be shared on reasonable request to the corresponding author.
