# Supplementary material for: Regulatory B cells improve ventricular remodeling after myocardial infarction by modulating monocyte migration
Source: Basic Res Cardiol. 2021 Jul 24;116(1):46. doi: 10.1007/s00395-021-00886-4 (PMC8310480; doi:10.1007/s00395-021-00886-4)
Supplement: Supplementary file 1 — Supplementary file1 (DOCX 4158 KB) [file 395_2021_886_MOESM1_ESM.docx]

**Supplementary material for**

**Regulatory B cells improve ventricular remodeling after myocardial infarction by modulating monocyte migration**

***Short title:*** Bregs improve ventricular remodeling after MI

Jiao Jiao^1,2^^#^·Shujie He^1,2^^#^·Yiqiu Wang^3#^·Yuzhi Lu^1,2^·Muyang Gu^1,2^·Dan Li^1,2^·Tingting Tang^1,2^·Shaofang Nie^1,2^·Min Zhang^1,2^·Bingjie Lv^1,2^·Jingyong Li^1,2^·Ni Xia^1,2^*·Xiang Cheng ^1,2^*

^1^Department of Cardiology, Union Hospital, Tongji Medical College, Huazhong University of Science and Technology, Wuhan 430022, China.

^2^Key Laboratory for Biological Targeted Therapy of Education Ministry and Hubei Province, Union Hospital, Tongji Medical College, Huazhong University of Science and Technology, Wuhan 430022, China.

^3^Department of Pediatrics, Union Hospital, Tongji Medical College, Huazhong University of Science and Technology, Wuhan, 430022, China.

^#^ These authors contributed equally to this paper.

***Addresses Correspondence to：**

| Dr. Xiang Cheng  Department of Cardiology, Union Hospital  Tongji Medical College of Huazhong University of Science and Technology  Key Laboratory for Biological Targeted Therapy of Education Ministry and Hubei Province  1277 Jiefang Road, Jianghan District  Wuhan 430022  China  Tel: 86-27-85726095  Email: nathancx@hust.edu.cn | Dr. Ni Xia  Department of Cardiology, Union Hospital  Tongji Medical College of Huazhong University of Science and Technology  Key Laboratory for Biological Targeted Therapy of Education Ministry and Hubei Province  1277 Jiefang Road, Jianghan District  Wuhan 430022  China  Tel: 86-27-85726011  Email: nixiaunion@163.com |
| --- | --- |

**Supplemental Methods**

**Echocardiographic analysis of cardiac function**

At day 28 post-MI, transthoracic echocardiography was performed using a Vevo 1100 high-resolution microimaging system (VisualSonics, Canada) by an experienced technician who was blinded to the treatment groups. The mice were anesthetized with 1.5% isoflurane and two-dimensional echocardiographic views of the mid-ventricular short axis and parasternal long axes were obtained. Left ventricular ejection fraction, left ventricular fractional shortening, left ventricular end-diastolic dimension (LVEDD) and left ventricular end-systolic dimension (LVESD) were calculated from digital images by using a standard formula as previously described [3, 4]. The heart rates of echocardiography data are shown in Supplementary Fig. 11.

**Scar size and fibrosis assessment**

The scar size and extent of fibrosis after 28 days were measured using Masson trichrome staining (Biossci Biotechnology, China). The scar size was calculated as (epicardial infarct ratio + endocardial infarct ratio)/2 × 100% [5]. For the assessment of fibrosis, we randomly selected five fields (200× magnification) in the peri-infarct zone and calculated the collagen volume fraction as the ratio of the blue-stained (collagen) area to the total tissue area using Image-Pro Plus software (Media Cybernetics Inc., USA) [6, 7].

**Cell isolation**

The Bregs were purified from the spleen of healthy mice using two different protocols. The first protocol was performed in three steps using a regulatory B cell isolation kit (Miltenyi Biotec, Germany) according to the manufacturer’s instructions. In short, B cells were isolated from the spleens of donor mice by negative selection. The pre-enriched B cells were stimulated with lipopolysaccharide (LPS, 10 μg/ml; Sigma-Aldrich, USA) for 48 h in culture and phorbol myristate acetate (PMA, 50 ng/ml, Sigma-Aldrich, USA) and ionomycin (500 ng/ml, Enzo, USA) were added during the last 5 h to induce IL-10 secretion. Subsequently, the viable IL-10 producing cells were specifically isolated by using the Cytokine Secretion Assay technology. An IL-10 specific Catch Reagent was attached to the cell surface and the cells were incubated in medium for 45 min at 37 ℃ to increase IL-10 secretion. These cells were then labeled with a second IL-10 specific antibody, the Regulatory B Cell Detection Antibody conjugated to PE. The IL-10-secreting cells were then magnetically labeled with Anti-PE specific MicroBeads and were enriched over magnetic-activated cell sorting (MACS) columns placed in the magnetic field of a MACS Separator. The purities of the isolated IL-10^+^ and IL-10^-^ B cell populations were routinely more than 90%. Another scheme for generating Bregs was consistent with published protocols [1, 2, 8]. Briefly, B cells from IL-10-GFP knock-in mice were enriched by positive selection using CD19 microbeads (Miltenyi Biotec, Germany), and purified B cells were cultured with LPS (10 μg/ml; Sigma-Aldrich, USA) for 48 h. Post-stimulation, the cells were subjected to fluorescence-activated cell sorting (FACS, BD Bioscience, USA), and CD19^+^GFP^+^ cells were selected.

Monocytes were positively selected from splenocytes of mice with CD11b microbeads (Miltenyi Biotec, Germany) according to the manufacturer’s instructions.

**Tracing experiment**

CD45.2^+^ mice received intravenous injection of 5 × 10^6^ Bregs obtained by MACS from the spleen of CD45.1^+^ mice after MI. Then, the heart, peripheral blood, spleen and bone marrow of the recipients were harvested 1 day, 3 days, 7 days or 14 days later, and the transferred cells were analyzed by flow cytometry.

**Flow cytometric analysis**

The heart, spleen, blood and bone marrow were collected post-MI, and single-cell suspensions were prepared for ﬂow cytometric analysis. In brief, hearts were minced and digested in 0.1% collagenase B solution (Roche Diagnostics GmbH, Germany) as previously described. Single cell suspensions were prepared by filtering through a 40 μm cell strainer (BD Bioscience, USA) and then leukocyte-enriched fractions were purified using 37/70% Percoll (GE Healthcare, USA) [4]. Spleens were excised, homogenized, and then passed through a 70 μm nylon mesh in phosphate buffered saline (PBS). Blood was collected and diluted in PBS. Bone marrow was prepared from the femur and processed with Hanks’ balanced salt solution. Then the mononuclear cells of the above tissues were isolated by Ficoll density gradient (Sigma-Aldrich, USA).

To detect the purities of the isolated Bregs and control B cells, the cells were first stained with APC anti-CD19 (BioLegend, USA) for 30 minutes and washed with PBS. Then the cells were fixed, permeabilized, and stained with PE anti-IL-10 (BioLegend, USA).

To detect the number of cardiac infiltrating T cell subsets, the harvested cells were stained with FITC anti-CD45, PE/Cy5 anti-CD11b, APC/Cy7 anti-CD3, PE/Cy7 anti-CD4 (all from BioLegend, USA) for 30 minutes and washed with PBS. Then the cells were fixed, permeabilized, and stained with PE anti-Foxp3 (eBioscience, USA).

To detect the number of cardiac infiltrating neutrophils, the accumulation of macrophages and the recruitment and mobilization of monocytes, the cells were labeled with FITC anti-CD45, PE/Cy5 anti-CD11b, APC/Cy7 anti-Ly6G, PE anti-F4/80, APC anti-CD206, PE/Cy7 anti-Ly6C (all from BioLegend, USA). Brilliant Violet 421 anti-CCR2 (BioLegend, USA) was used to measure the CCR2 expression in monocytes from the spleen, blood and bone marrow.

For tracking the adoptively transferred Bregs in vivo, the mononuclear cells in the heart, spleen, peripheral blood and bone marrow were stained with FITC anti-CD45.2, APC anti-CD45.1, and Brilliant Violet 605 anti-CD19 (all from BioLegend, USA). Flow cytometry was performed using a FACS Calibur (BD Bioscience, USA) and the results were analyzed by FlowJo software (Tree Star, USA).

**Cell culture and transwell experiment**

Splenic monocytes (1 × 10^6^/ml) were purified and cultured alone or with isolated Bregs or control B cells (1 × 10^6^/ml) for 24 h. Transwell experiments were performed in 24-well plates (0.4 μm pore size, Corning Life Sciences, USA) with monocytes in the lower chamber and Bregs in the insert. For neutralization experiments, the neutralizing antibody against IL-10 (20μg/ml, R&D, USA) or isotype control antibody was added to the co-culture system. The cells were collected and monocyte CCR2 expression was assessed by flow cytometry as demonstrated above.

**RT-qPCR**

Total RNA was extracted from freshly isolated monocytes, Bregs and control B cells with Trizol Isolation Reagent (Takara, Japan) and reverse transcribed into cDNA using the PrimeScript RT Reagent Kit (Takara, Japan) according to the manufacturer’s instructions. Reverse transcription polymerase chain reactions were prepared with sequence-specific primers and SYBR Green Master Mix (Takara, Japan) in a 10 μl volume. RT-qPCR was performed using a CFX96 Real-Time PCR Detection System (Bio-Rad, USA) and each reaction was performed in duplicate. Data from each sample were standardized with GAPDH using the 2^-ΔΔCT^ method. Primer sequences are presented in Supplemental table 1.

**ELISA**

The concentrations of C-C motif chemokine ligand 2 (CCL2) and C-C motif chemokine ligand 7 (CCL7) in the mouse plasma were analyzed using commercial ELISA kits (eBioscience, USA) according to the manufacturer’s instructions.

To detect the expression of IL-10, spleens were excised and homogenized in PBS (1 ml), the femurs were flushed with PBS (1 ml) and the perfusate collected. After centrifugation (300g for 10 min), the supernatants were analyzed using an IL-10 ELISA kit (NeoBioscience, China).

To evaluated the IL-10, TGFβ1 and IL-35 protein expression, splenic Bregs and control B cells were purified and cultured (1 × 10^6^/ml) for 24 h under the stimulation of LPS (10 μg/ml, Sigma-Aldrich, USA), and PMA (50 ng/ml, Sigma-Aldrich, USA) and ionomycin (500 ng/ml, Enzo, USA) were added during the last 5 h. Then the culture supernatants were collected and analyzed by IL-10 (NeoBioscience, China), TGFβ1 (NeoBioscience, China) and IL-35 (BioLegend, USA) ELISA kits according to the manufacturer’s instructions.

**Reference**

1. Bodhankar S, Chen Y, Vandenbark AA, Murphy SJ, Offner H (2013) IL-10-producing B-cells limit CNS inflammation and infarct volume in experimental stroke. Metab Brain Dis 28:375-386 doi:10.1007/s11011-013-9413-3

2. Bodhankar S, Chen Y, Vandenbark AA, Murphy SJ, Offner H (2014) Treatment of experimental stroke with IL-10-producing B-cells reduces infarct size and peripheral and CNS inflammation in wild-type B-cell-sufficient mice. Metab Brain Dis 29:59-73 doi:10.1007/s11011-013-9474-3

3. Li Y, Garson CD, Xu Y, Beyers RJ, Epstein FH, French BA, Hossack JA (2007) Quantification and MRI Validation of Regional Contractile Dysfunction in Mice Post Myocardial Infarction Using High Resolution Ultrasound. Ultrasound Med Biol 33:894-904 doi:10.1016/j.ultrasmedbio.2006.12.008

4. Liao YH, Xia N, Zhou SF, Tang TT, Yan XX, Lv BJ, Nie SF, Wang J, Iwakura Y, Xiao H, Yuan J, Jevallee H, Wei F, Shi GP, Cheng X (2012) Interleukin-17A contributes to myocardial ischemia/reperfusion injury by regulating cardiomyocyte apoptosis and neutrophil infiltration. J Am Coll Cardiol 59:420-429 doi:10.1016/j.jacc.2011.10.863

5. Takagawa J, Zhang Y, Wong ML, Sievers RE, Kapasi NK, Wang Y, Yeghiazarians Y, Lee RJ, Grossman W, Springer ML (2007) Myocardial infarct size measurement in the mouse chronic infarction model: comparison of area- and length-based approaches. J Appl Physiol (1985) 102:2104-2111 doi:10.1152/japplphysiol.00033.2007

6. Tang TT, Li YY, Li JJ, Wang K, Han Y, Dong WY, Zhu ZF, Xia N, Nie SF, Zhang M, Zeng ZP, Lv BJ, Jiao J, Liu H, Xian ZS, Yang XP, Hu Y, Liao YH, Wang Q, Tu X, Mallat Z, Huang Y, Shi GP, Cheng X (2018) Liver-heart crosstalk controls IL-22 activity in cardiac protection after myocardial infarction. Theranostics 8:4552-4562 doi:10.7150/thno.24723

7. Tang TT, Yuan J, Zhu ZF, Zhang WC, Xiao H, Xia N, Yan XX, Nie SF, Liu J, Zhou SF, Li JJ, Yao R, Liao MY, Tu X, Liao YH, Cheng X (2012) Regulatory T cells ameliorate cardiac remodeling after myocardial infarction. Basic Res Cardiol 107:232 doi:10.1007/s00395-011-0232-6

8. Zhang J, Benedek G, Bodhankar S, Lapato A, Vandenbark AA, Offner H (2015) IL-10 producing B cells partially restore E2-mediated protection against EAE in PD-L1 deficient mice. J Neuroimmunol 285:129-136 doi:10.1016/j.jneuroim.2015.06.002

**Supplemental Figures**

**
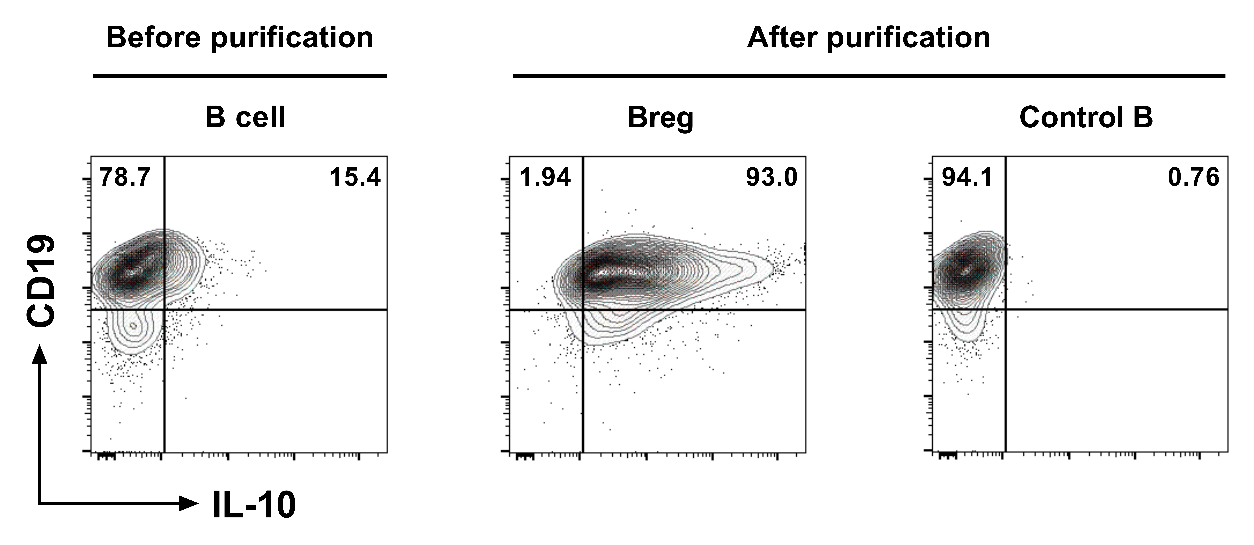
**

**Supplementary Fig. 1 The purity of Bregs after isolation**

Representative flow cytometric images of CD19^+^IL-10^+^ Bregs before and after purification.

**
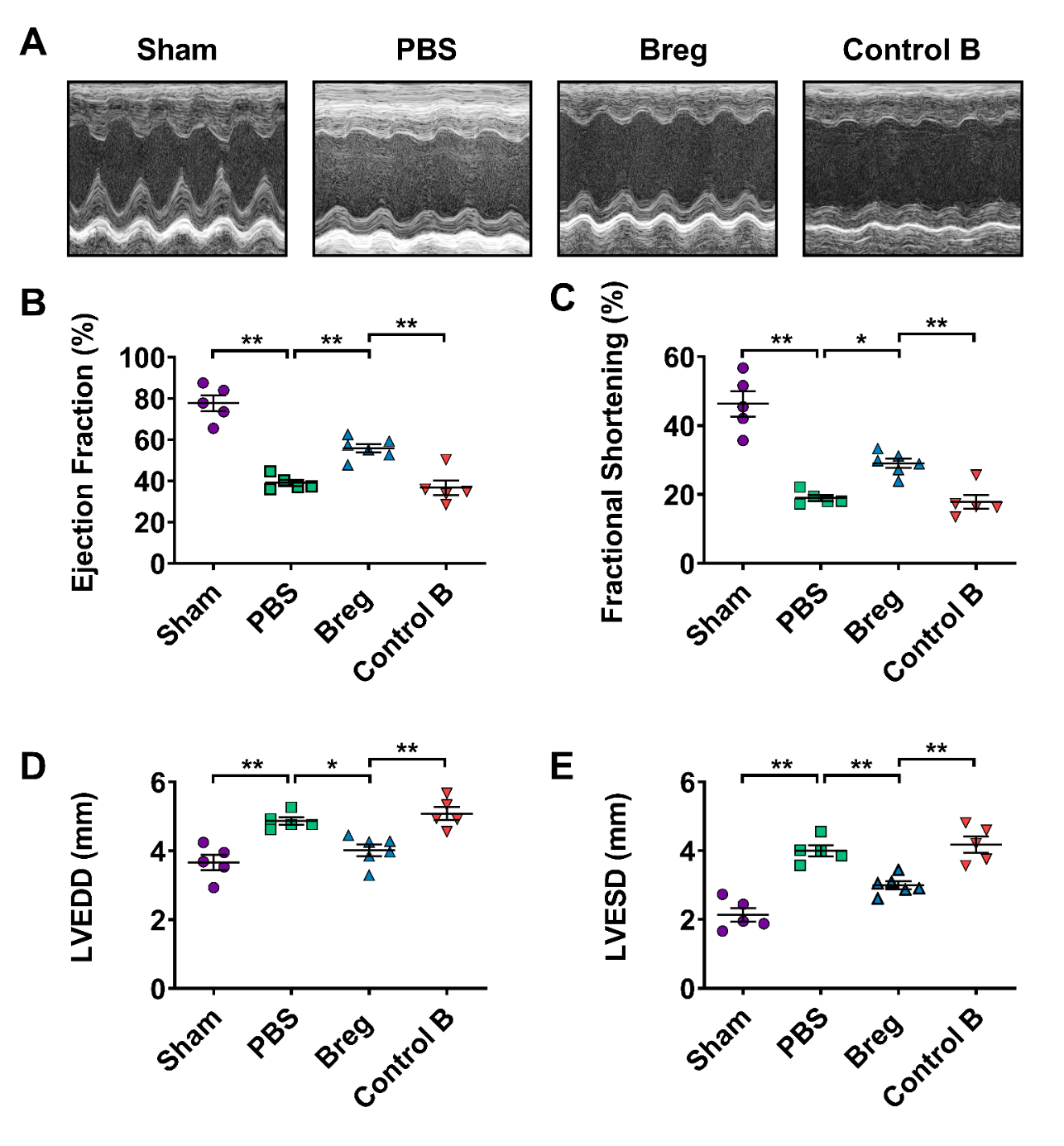
**

**Supplementary Fig. 2 Adoptive transfer of Bregs sorted by FACS improves cardiac function after MI**

**a** Representative M‐mode echocardiographic images of the left ventricle 28 days after MI.

**b-e** Quantification of ejection fraction (**b**), fractional shortening (**c**), LVEDD (**d**) and LVESD (**e**) at day 28 after MI. n = 5-6 per group.

Data are expressed as means ± SEM. **P* < 0.05, ***P* < 0.01. Data in **b-e** were analyzed by one-way ANOVA, followed by Tukey’s post hoc test. Sham: sham-operated group, PBS: MI mice that received phosphate buffered saline, Breg: MI mice that received regulatory B cells, Control B: MI mice that received control B cells, LVEDD: left ventricular end-diastolic dimension, LVESD: left ventricular end-systolic dimension.


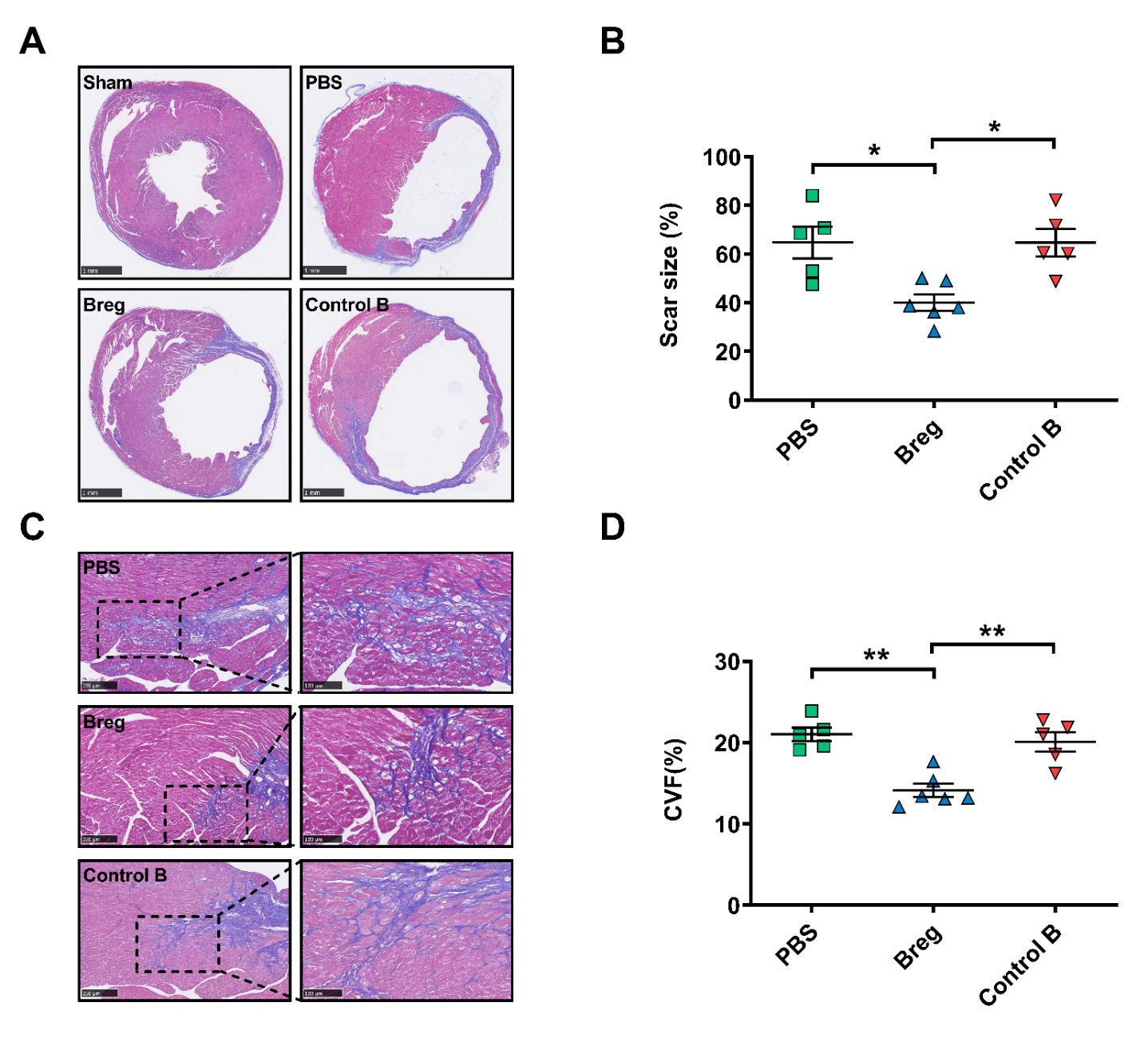


**Supplementary Fig. 3 Adoptive transfer of Bregs sorted by FACS reduces scar size and fibrosis after MI**

**a** Representative photomicrographs of scar size evaluated by Masson trichrome staining 28 days after MI. Scale bar: 1 mm.

**b** Quantitative analysis of scar size by Masson trichrome staining at day 28 after MI. n = 5-6 per group.

**c** Representative images indicating collagen deposition (blue) evaluated by Masson trichrome staining at day 28 after MI. Scale bar: 250 μm (left) or 100 μm (right).

**d** Fibrosis assessed by CVF in the peri-infarct zone was compared among the different treatments at day 28 after MI. n = 5-6 per group.

Data are expressed as means ± SEM. **P* < 0.05, ***P* < 0.01. Data in **b** and **d** were analyzed by one-way ANOVA, followed by Tukey’s post hoc test. Sham: sham-operated group, PBS: MI mice that received phosphate buffered saline, Breg: MI mice that received regulatory B cells, Control B: MI mice that received control B cells, CVF: collagen volume fraction.

**
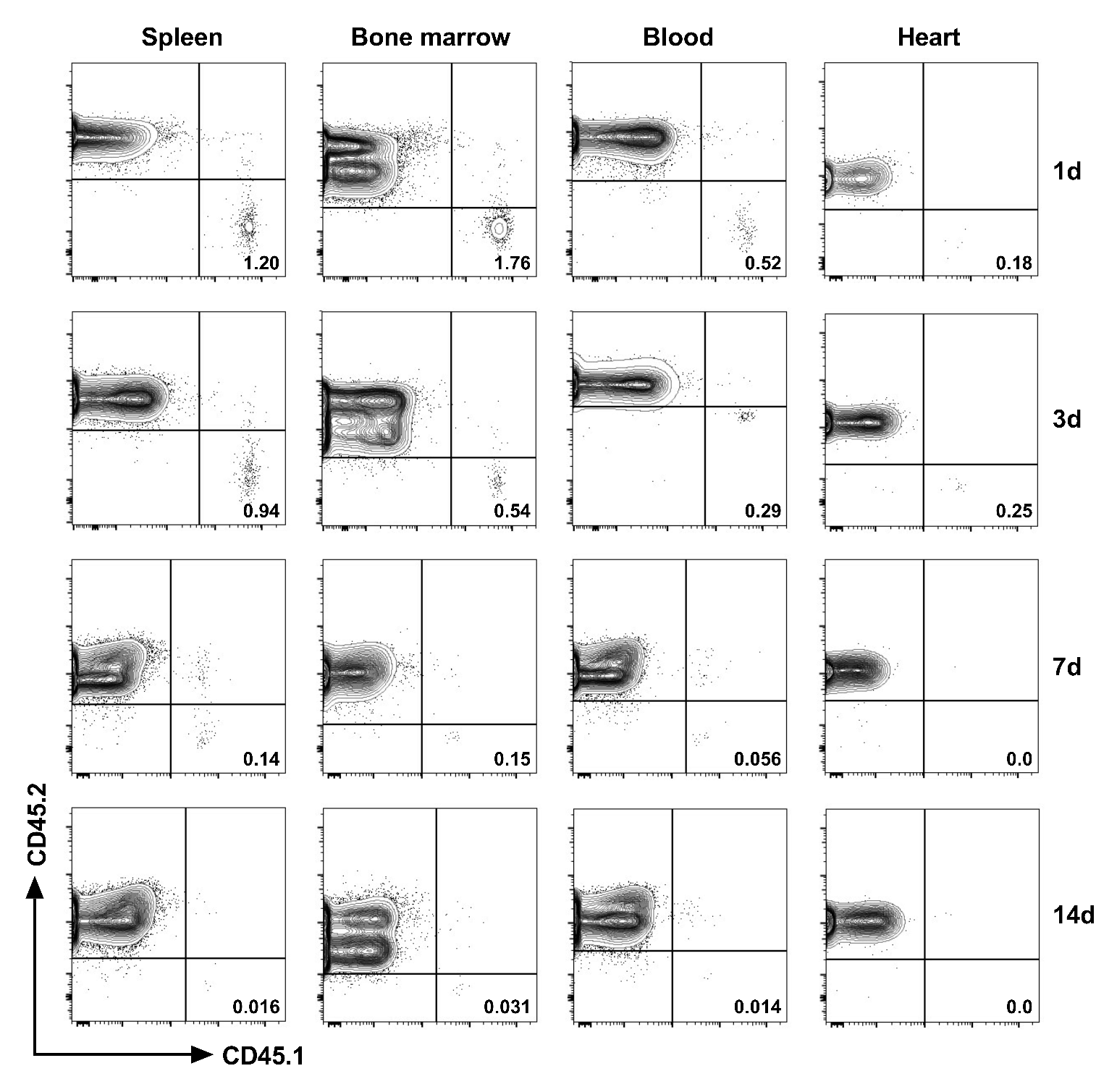
**

**Supplementary Fig. 4 Adoptively transferred Bregs primarily present in the spleen and bone marrow**

Representative flow cytometric images of adoptively transferred CD45.1^+^ Bregs in the spleen, bone marrow, blood and heart 1 day, 3 days, 7 days and 14 days after MI. Three independent experiments were performed for each time point. 1d: 1 day post-MI, 3d: 3 days post-MI, 7d: 7 days post-MI, 14d: 14 days post-MI.


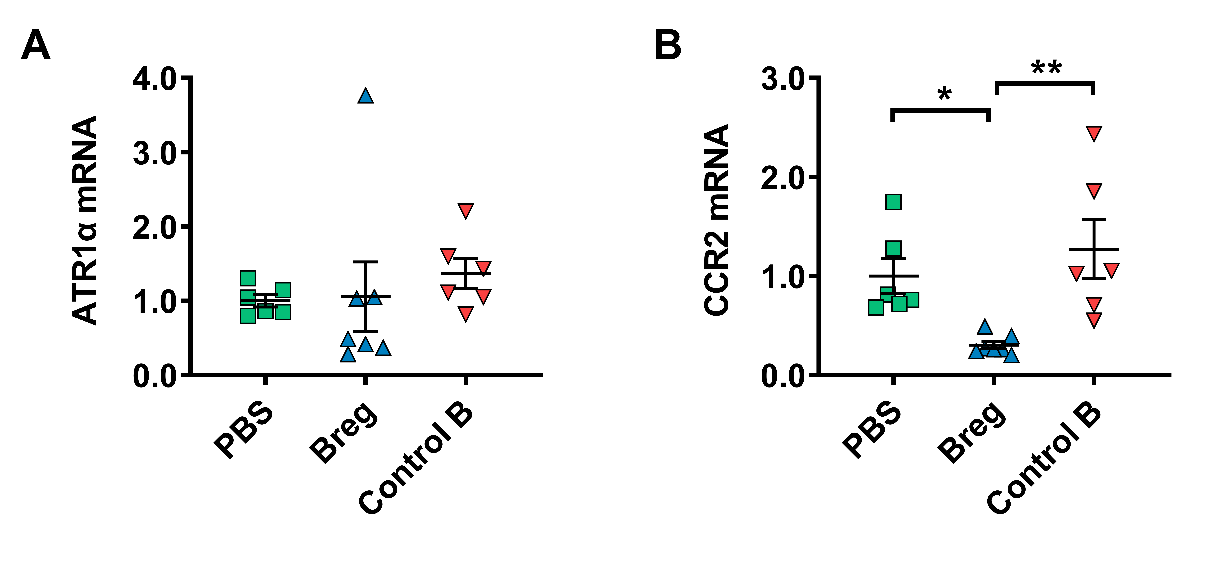


**Supplementary Fig. 5 Bregs impair the expression of CCR2 in the splenic monocytes after MI**

**a, b** PBS, Bregs or control B cells were injected intravenously after ligation of the coronary artery and monocytes were collected from the spleen 1 day after MI. The gene expression of ATR1 (**a**), CCR2 (**b**) was analyzed by RT-qPCR. n = 6-7 per group.

Data are expressed as means ± SEM. **P* < 0.05, ***P* < 0.01. Data in **a** and **b** were analyzed by Kruskal-Wallis test with Dunn’s multiple comparisons test. PBS: MI mice that received phosphate buffered saline, Breg: MI mice that received regulatory B cells, Control B: MI mice that received control B cells, AT1Rα: angiotensin II type 1 receptor α.


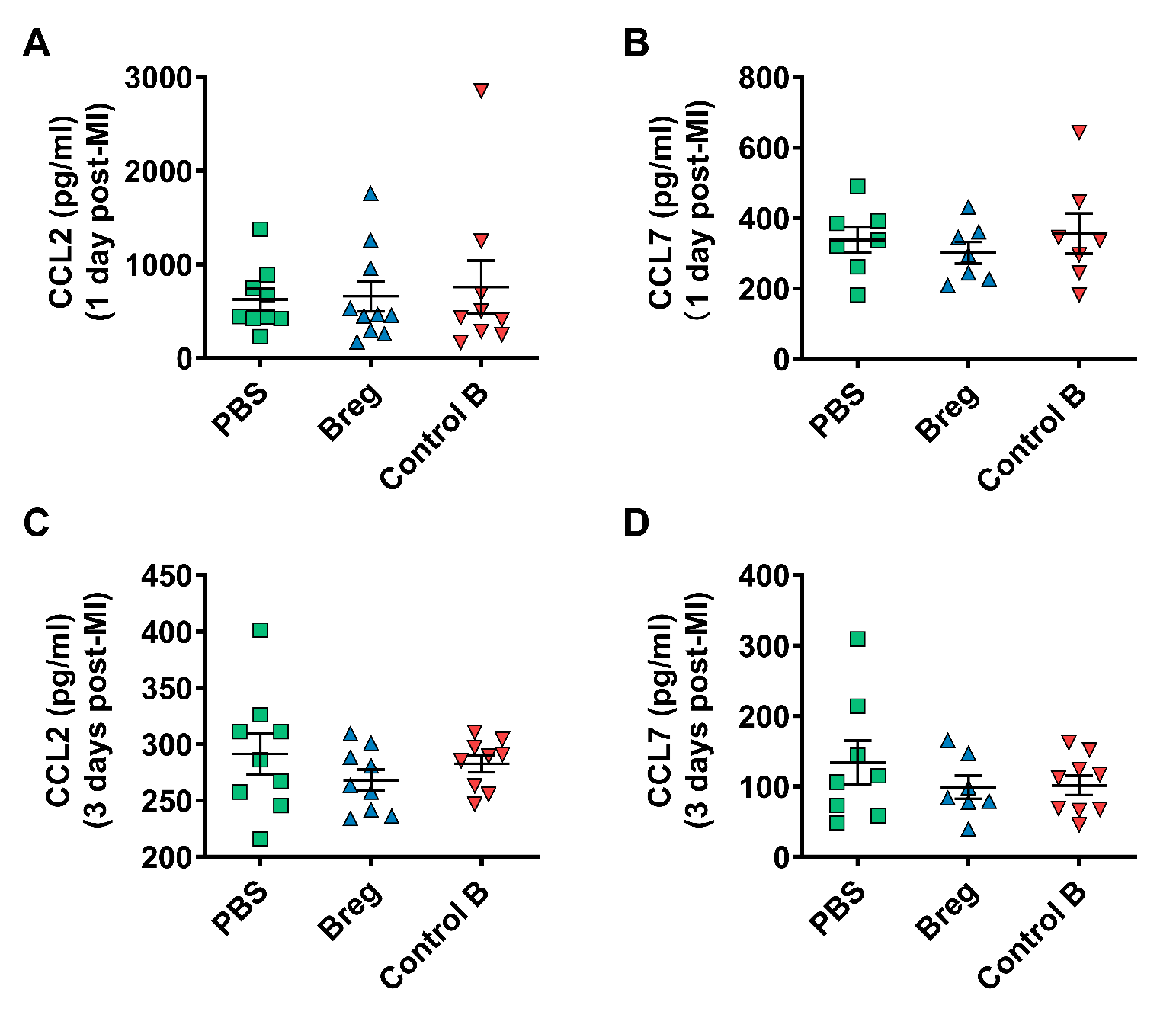


**Supplementary Fig. 6 The levels of CCL2 and CCL7 in the peripheral blood do not change after adoptive transfer of Bregs post-MI**

**a, b** The levels of CCL2 (**a**) and CCL7 (**b**) in the serum were detected by ELISA 1 day after MI. n = 7-10 per group.

**c, d** The levels of CCL2 (**c**) and CCL7 (**d**) in the serum were detected by ELISA 3 days after MI. n = 7-10 per group.

Data are expressed as means ± SEM. Data in **a** and **c** were analyzed by Kruskal-Wallis test with Dunn’s multiple comparisons test. Data in **b** and **d** were analyzed by one-way ANOVA, followed by Tukey’s post hoc test. PBS: MI mice that received phosphate buffered saline, Breg: MI mice that received regulatory B cells, Control B: MI mice that received control B cells.

**
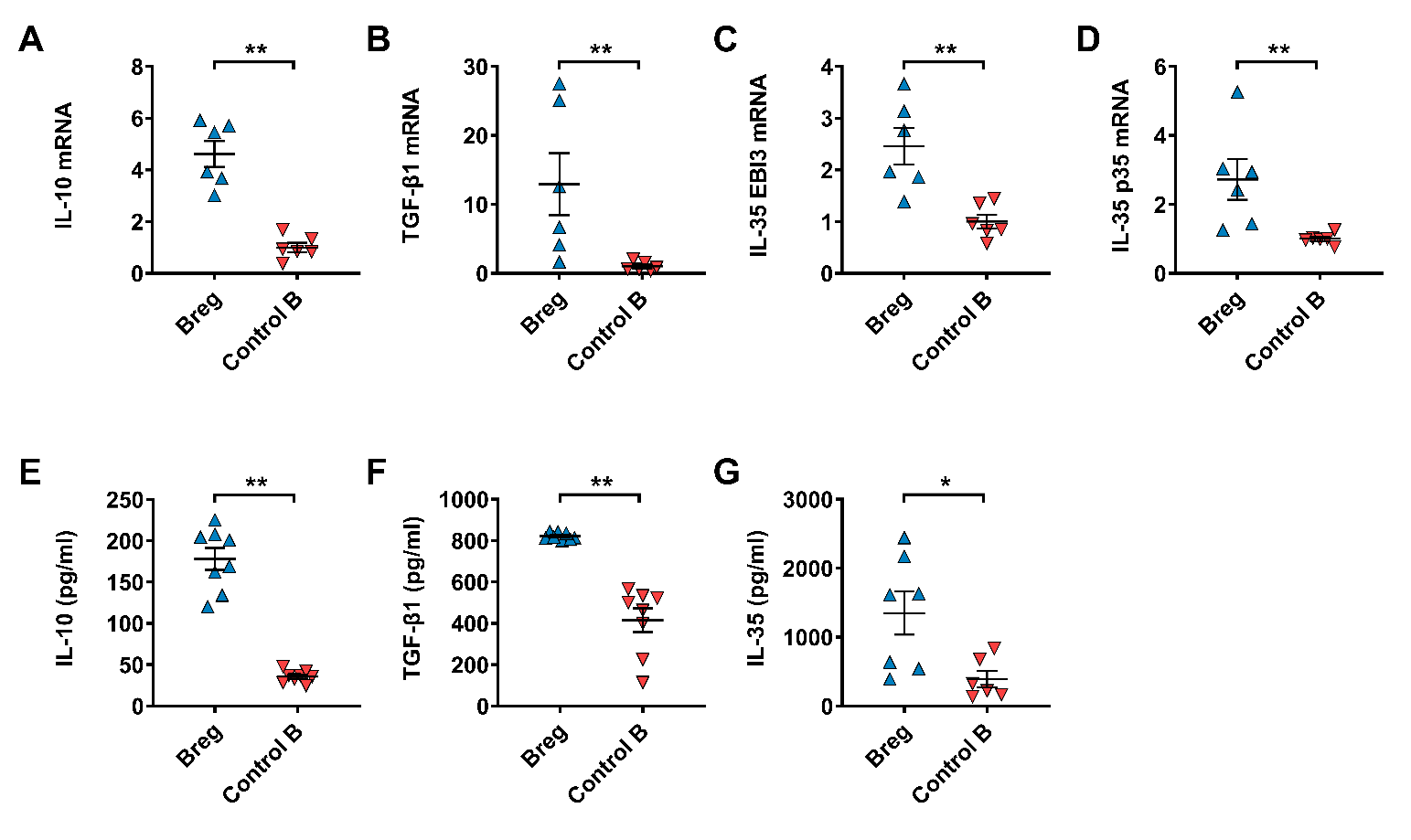
**

**Supplementary Fig. 7 Bregs express higher levels of IL-10, TGF-β1 and IL-35 than control B cells**

**a-d** Bregs and control B cells were sorted from the spleen. The gene expression of IL-10 (**a**), TGF-β1 (**b**), IL-35 EBI3 (**c**) and IL-35 p35 (**d**) was measured by RT-qPCR. n = 6 per group.

**e-g** The purified Bregs and control B cells were cultured for 24 h under the stimulation of LPS and then the protein expression of IL-10 (**e**), TGF-β1 (**f**), and IL-35 (**g**) in the supernatant was measured by ELISA. n = 6-8 per group.

Data are expressed as means ± SEM. **P* < 0.05, ***P* < 0.01. Data in **a**, **b**, **d** and **e-g** were analyzed by Mann-Whitney U-test. Data in **c** were analyzed by unpaired Student’s *t* test.

**
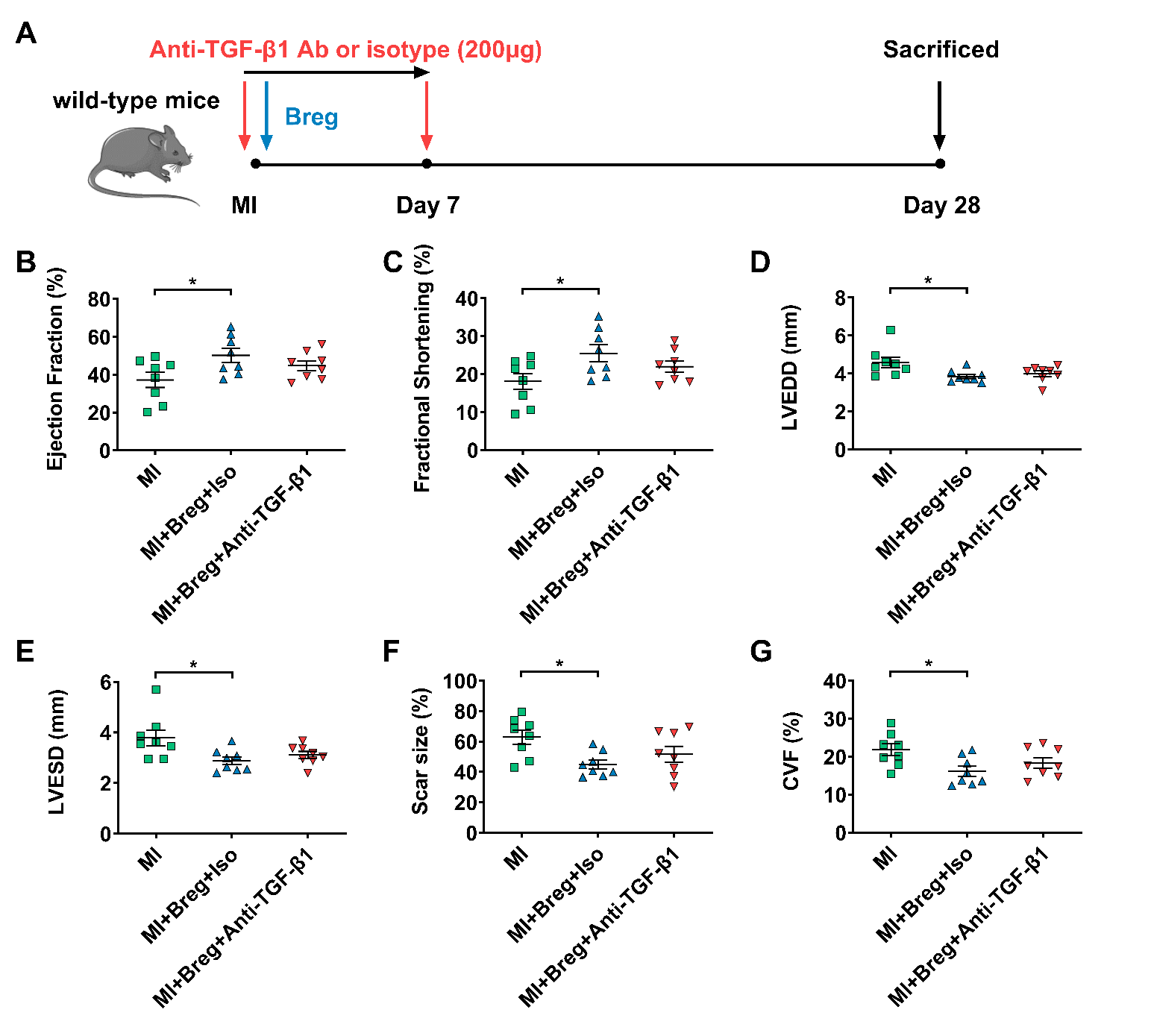
**

**Supplementary Fig. 8 The protective effect of Bregs against MI is independent of TGF-β1**

**a** Experimental procedures and timeline of surgery and treatment are shown. MI mice were administered with Bregs along with the anti-TGF-β1 antibody or isotype control antibody.

**b-e** Ejection fraction (**b**), fractional shortening (**c**), LVEDD (**d**) and LVESD (**e**) were assessed using echocardiography 28 days after MI. n = 8 per group.

**f, g** Scar size (**f**) and CVF (**g**) were measured by Masson trichrome staining 28 days after MI. n = 8 per group.

Data are expressed as means ± SEM. **P* < 0.05. Data in **b-g** were analyzed by one-way ANOVA, followed by Tukey’s post hoc test. MI: MI mice control group, MI + Breg + Iso: MI mice that received regulatory B cells along with the isotype control antibody, MI + Breg + Anti-TGF-β1: MI mice that received regulatory B cells along with the anti-TGF-β1 antibody, Ab: antibody, LVEDD: left ventricular end-diastolic dimension, LVESD: left ventricular end-systolic dimension, CVF: collagen volume fraction.


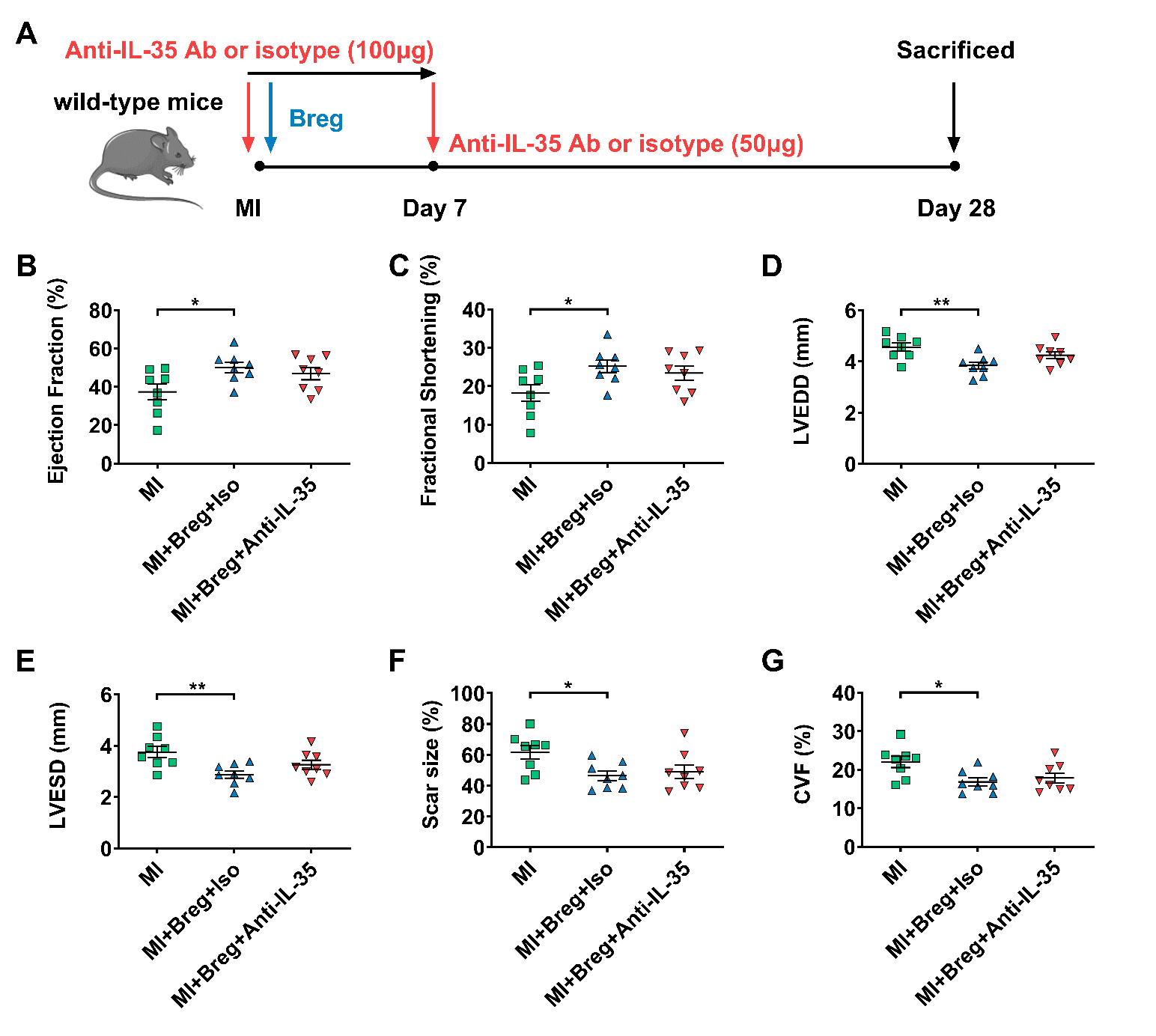


**Supplementary Fig. 9 The protective effect of Bregs against MI does not rely on IL-35**

**a** Experimental procedures and timeline of surgery and treatment are shown. MI mice were administered with Bregs along with the anti-IL-35 antibody or isotype control antibody.

**b-e** Ejection fraction (**b**), fractional shortening (**c**), LVEDD (**d**) and LVESD (**e**) were assessed using echocardiography 28 days after MI. n = 8 per group.

**f, g** Scar size (**f**) and CVF (**g**) were measured by Masson trichrome staining 28 days after MI. n = 8 per group.

Data are expressed as means ± SEM. **P* < 0.05, ***P* < 0.01. Data in **b-g** were analyzed by one-way ANOVA, followed by Tukey’s post hoc test. MI: MI mice control group, MI + Breg + Iso: MI mice that received regulatory B cells along with the isotype control antibody, MI + Breg + Anti-IL-35: MI mice that received regulatory B cells along with the anti-IL-35 antibody, Ab: antibody, LVEDD: left ventricular end-diastolic dimension, LVESD: left ventricular end-systolic dimension, CVF: collagen volume fraction.

**
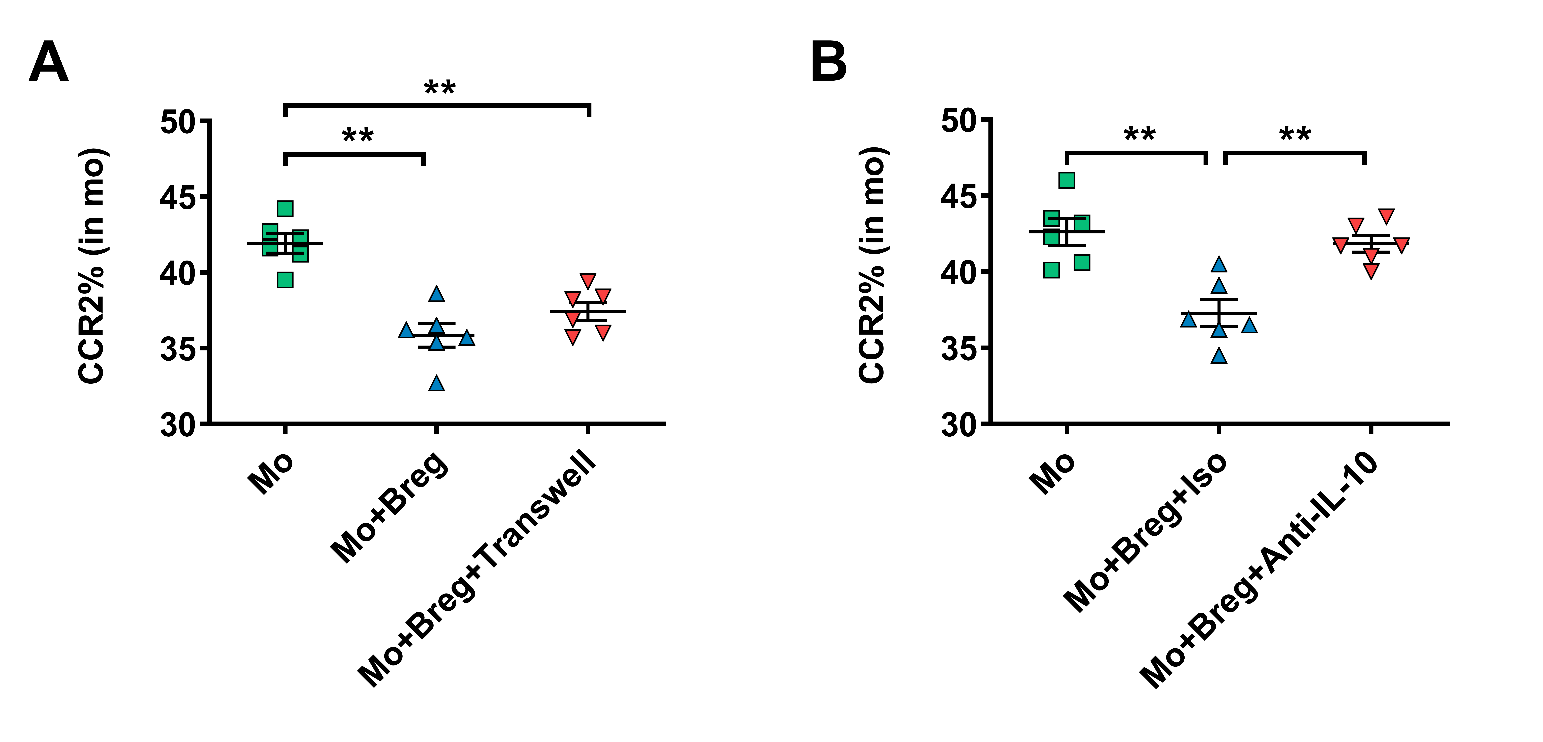
**

**Supplementary Fig. 10 Anti-IL-10 antibody antagonizes the effect of Bregs on monocytes *in vitro***

**a** Splenic monocytes were co-cultured with Bregs in the same well or cultured separately in a transwell system, with Bregs in the insert and monocytes in the lower chamber. After 1 day, CCR2 in monocytes was measured by flow cytometry. n = 6 per group.

**b** Monocytes sorted from the spleen were co-cultured with Bregs in the presence of the anti-IL-10 antibody or isotype control antibody. After 1 day, CCR2 in monocytes was measured by flow cytometry. n = 6 per group.

Data are expressed as means ± SEM. ***P* < 0.01. Data in **a** and **b** were analyzed by one-way ANOVA, followed by Tukey’s post hoc test. Mo: monocytes cultured alone, Mo + Breg: monocytes co-cultured with regulatory B cells, Mo + Breg + Transwell: monocytes co-cultured with regulatory B cells in a transwell system, Mo + Breg + Iso: monocytes co-cultured with regulatory B cells in the presence of the isotype control antibody, Mo + Breg + anti-IL-10: monocytes co-cultured with regulatory B cells in the presence of the anti-IL-10 antibody.

**
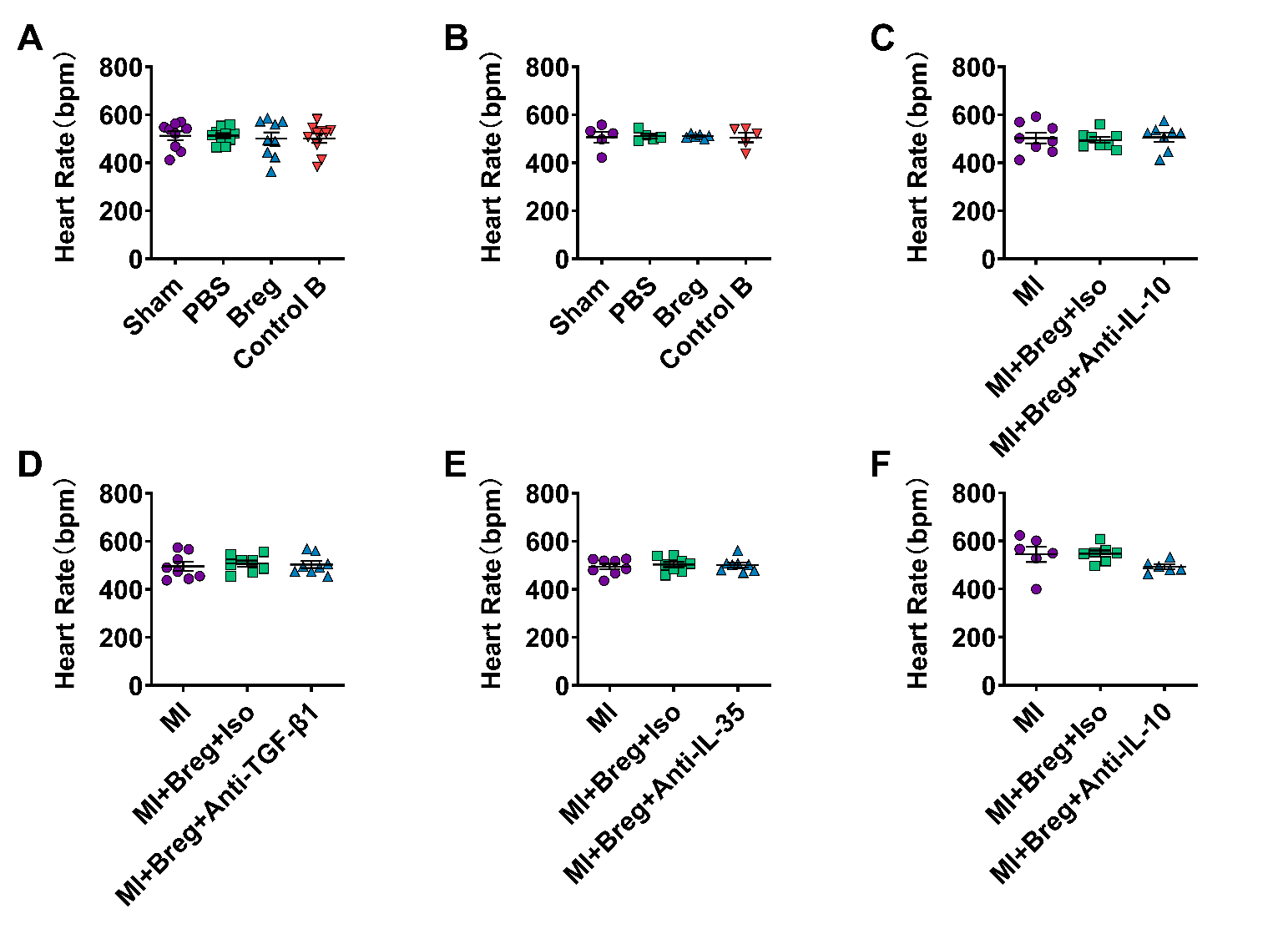
**

**Supplementary Fig 11 The heart rates of mice among the different treatment groups**

**a** MI mice were administered with Bregs isolated by MACS and heart rates were assessed using echocardiography 28 days after MI. n = 9-10 per group.

**b** MI mice were administered with Bregs isolated by FACS and heart rates were assessed using echocardiography 28 days after MI. n = 5-6 per group.

**c** MI mice were administered with Bregs along with the anti-IL-10 antibody or isotype control antibody. Heart rates were assessed using echocardiography 28 days after MI. n = 8 per group.

**d** MI mice were administered with Bregs along with the anti-TGF-β1 antibody or isotype control antibody. Heart rates were assessed using echocardiography 28 days after MI. n = 8 per group.

**e** MI mice were administered with Bregs along with the anti-IL-35 antibody or isotype control antibody. Heart rates were assessed using echocardiography 28 days after MI. n = 8 per group.

**f** IL-10 KO MI mice were administered with Bregs along with the anti-IL-10 antibody or isotype control antibody. Heart rates were assessed using echocardiography 28 days after MI. n = 6-7 per group.

Data are expressed as means ± SEM. Data in **a** and **c-e** were analyzed by one-way ANOVA, followed by Tukey’s post hoc test. Data in **b** and **f** were analyzed by Kruskal-Wallis test with Dunn’s multiple comparisons test. Sham: sham-operated group, PBS: MI mice that received phosphate buffered saline, Breg: MI mice that received regulatory B cells, Control B: MI mice that received control B cells, MI: MI mice control group, MI + Breg + Iso: MI mice that received regulatory B cells along with the isotype control antibody, MI + Breg + Anti-IL-10: MI mice that received regulatory B cells along with the anti-IL-10 antibody, MI + Breg + Anti-TGF-β1: MI mice that received regulatory B cells along with the anti-TGF-β1 antibody, MI + Breg + Anti-IL-35: MI mice that received regulatory B cells along with the anti-IL-35 antibody.

**Supplementary Table 1 List of the primer sequences for RT-qPCR in this study**

| **genes** | **Forward (5’-3’)** | **Reverse (5’-3’)** |
| --- | --- | --- |
| *CCR2* | ATCCACGGCATACTATCAACATC | TCGTAGTCATACGGTGTGGTG |
| *ATR1* | TTGTCCACCCGATGAAGTC | TCCTATGGGGAGCGTTGA |
| *IL-10* | CTTACTGACTGGCATGAGGATCA | GCAGCTCTAGGAGCATGTGG |
| *TGF-β1* | CTCCCGTGGCTTCTAGTGC | GCCTTAGTTTGGACAGGATCTG |
| *IL-35 EBI3* | CGGTGCCCTACATGCTAAAT | GCGGAGTCGGTACTTGAGAG |
| *IL-35 p35* | CATCGATGAGCTGATGCAGT | CAGATAGCCCATCACCCTGT |
| *Gapdh* | GACGGCCAGGTCATCACTATTG | CCACAGGATTCCATACCCAAGA |
